# Enhanced memory despite severe sleep loss in *Drosophila insomniac* mutants

**DOI:** 10.1371/journal.pbio.3003076

**Published:** 2025-03-20

**Authors:** Sheng Huang, Chengji Piao, Zhiying Zhao, Christine B. Beuschel, Oriane Turrel, David Toppe, Stephan J. Sigrist

**Affiliations:** 1 Institute for Biology/Genetics, Freie Universität Berlin, Berlin, Germany; 2 NeuroCure Cluster of Excellence, Charité Universitätsmedizin, Berlin, Germany; Washington University, St.Louis, MO 63110, UNITED STATES OF AMERICA

## Abstract

Sleep is crucial for cognitive functions and life span across species. While sleep homeostasis and cognitive processes are linked through cellular and synaptic plasticity, the signaling pathways connecting them remain unclear. Here, we show that *Drosophila insomniac* (*inc*) short sleep mutants, which lack an adaptor protein for the autism-associated Cullin-3 ubiquitin ligase, exhibited enhanced Pavlovian aversive olfactory learning and memory, unlike other sleep mutants with normal or reduced memory. Through a genetic modifier screen, we found that a mild reduction of Protein Kinase A (PKA) signaling specifically rescued the sleep and longevity phenotypes of *inc* mutants. However, this reduction further increased their excessive memory and mushroom body overgrowth. Since *inc* mutants displayed higher PKA signaling, we propose that *inc* loss-of-function suppresses sleep via increased PKA activity, which also constrains the excessive memory of *inc* mutants. Our data identify a signaling cascade for balancing sleep and memory functions, and provide a plausible explanation for the sleep phenotypes of *inc* mutants, suggesting that memory hyperfunction can provoke sleep deficits.

## Abstract 2

Sleep is known to support memory. In this study, the authors identify an instance where memory hyperfunction may actually provoke sleep deficits. They find that the short-sleep *Drosophila* mutants, *insomniac* , has enhanced memory and that PKA signaling balances sleep and memory in these flies.

## Introduction

Sleep is a dynamic process conserved from invertebrates to mammals and humans [1–3]. The molecular, cellular and circuit mechanisms encoding sleep need and executing sleep behavior have been intensively studied [3–5]. Though sleep is thought to serve many purposes, it is often studied for its restorative roles, which are believed to optimize life span and cognition [6]. Indeed, acute- and long-term sleep loss can have adverse effects on both memory and survival [7–10], while efficient sleep protects against stress, aging and disease [8,11–13]. Recent studies suggest that multiple tissues and organs mediate the consequences of sleep loss, for example gut oxidative stress and peripheral tissue metabolic alterations [8,14,15]. However, the brain as a signaling hub integrates global information including those from peripheral tissues and circuits to tune the final behavioral output [12,16,17]. Plastic changes in molecular and circuit levels coupling and balancing sleep and memory are thus likely harnessed within core integration neurons and circuits controlling both behavioral processes [18–20]. However, how sleep is regulated to optimize memory formations and support survival remains to be further explored.

The fruitfly *Drosophila melanogaster* has long been used to study associative learning and memory [21]. Flies exhibit sleep states similar to mammals and humans, with reduced locomotion, increased arousal threshold, and compensatory sleep rebound upon sleep loss [22,23]. Similar to the mammalian hippocampus [24], the mushroom body as a high level integration center of the fly brain plays essential roles in both memory and sleep regulation [19,20,25–27]. Activating or silencing mushroom body intrinsic neurons (Kenyon cells) and output neurons can change the patterns of sleep and wakefulness [10,17,19,20,26,27]. Importantly, bidirectional genetic manipulations of the cyclic adenosine 3′, 5′-monophosphate (cAMP) and Protein Kinase A (PKA) signaling suggest that cAMP/PKA signaling negatively regulates sleep [19,28]. Interestingly, though cAMP/PKA signaling is indispensable for associative memory, excessive PKA activity can also suppress memory [29,30]. Conversely, reducing gene dose of PKA catalytic subunit Dc0 protects from age-associated memory decline in *Drosophila* [29,30]. In the rodent hippocampus, sleep loss reduces cAMP level and long-term potentiation and impairs cognitive functions [24]. While these findings highlight core cellular and molecular mechanisms linking memory formations and sleep regulation across animal models, it remains unclear whether and how cAMP/PKA signaling in the fly mushroom body controls the balance between memory function and sleep levels.

*Drosophila* has been exploited to identify a spectrum of evolutionarily conserved genes in regulating sleep [3,4]*.* Among them, the *insomniac* (*inc*) locus, encoding an adaptor protein for Cullin-3 E3 ligase-mediated ubiquitination, was identified in two independent genetic screens to be essential for promoting sleep [31,32]. Inc is evolutionarily conserved and its mouse orthologs can functionally restore the sleep of *inc* mutant flies [33]. Inc has strong expression in the mushroom body, and it is essential for proper development of the mushroom body circuit as well as for the execution of presynaptic homeostatic plasticity [34–36]. Furthermore, *inc* mutants are hypersensitive to oxidative stress and short-lived [11,13,31]. However, the cellular and molecular mechanisms by which Inc modulates Cullin-3 ligase to regulate sleep remain unknown.

In this study, we firstly demonstrate that *inc* mutants showed robustly increased performance in Pavlovian olfactory learning and memory, despite their severe sleep deficits. A genetic modifier sleep screen identified the PKA signaling pathway in specifically mediating the sleep deficits of *inc* mutants. Surprisingly, the excessive memory of *inc* mutants was further increased by *Dc0* heterozygosity, suggesting that *inc* loss-of-function (LOF) suppresses sleep through elevated PKA signaling, which in turn counterbalances and limits the excessive memory of *inc* mutants. Taken together, our data reveal higher memory performance despite severe sleep loss in *inc* mutants and illustrate how intrinsic PKA signaling modulation in *inc* mutants rebalances sleep and memory functions.

## Results

### *insomniac* (*inc*) short sleep mutants display increased performance in Pavlovian aversive olfactory learning and memory

**Fig 1:**
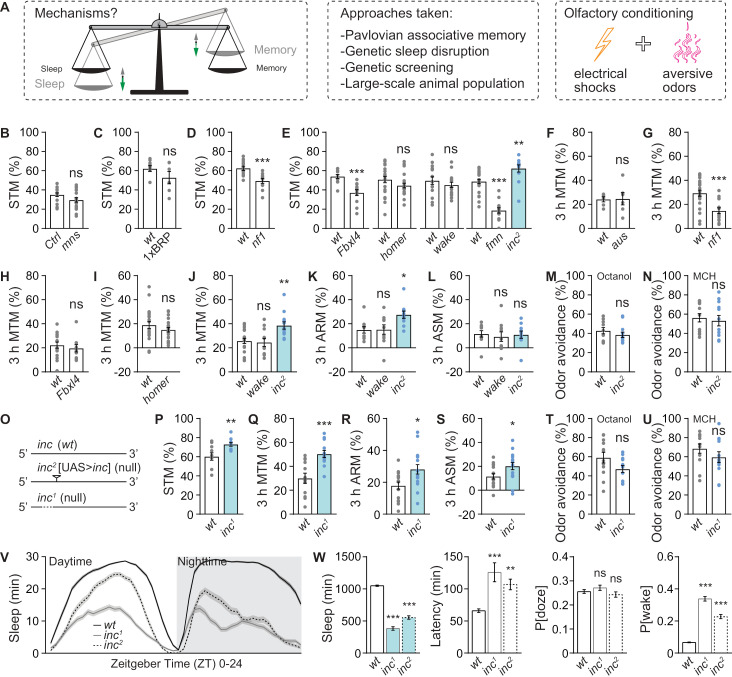
Olfactory learning and memory screen identified Inc protein as a memory suppressor. (**A**) Rationale and approaches for studying the molecular mechanisms for the balance between the amount of sleep and the strength of memory functions. (**B**–**E**) Olfactory short-term memory (STM, or learning) tested immediately after training for *Shaker mns* (**B**), 1xBRP (**C**), *nf1* (**D**), and *Fbxl4*, *homer*, *wake*, *fmn* and *inc*^*2*^ (**E**). *n* = 18 for *mns*, 6–9 for 1xBRP, 11–12 for *nf1*, 12 for *Fbxl4*, 22 for *homer*, 16 for *wake*, and 12–25 for *fmn* and *inc*^*2*^. (**F**–**J**) Olfactory middle-term memory (MTM) tested 3 h after training for *aus* (**F**), *nf1* (**G**), *Fbxl4* (**H**), *homer* (**I**), and *wake* and *inc*^*2*^ (**J**). *n* = 6 for *aus*, 14–22 for *nf1*, 10–16 for *Fbxl4*, 21–23 for *homer*, and 11–19 for *wake* and *inc*^*2*^. (**K** and **L**) Anesthesia-resistant memory (ARM) (**K**) and anesthesia-sensitive memory (ASM) (**L**) tested 3 h after training for *wake* and *inc*^*2*^. *n* = 10. (**M** and **N**) Odor avoidance of *inc*^*2*^ for octanol (**M**) and 4-methylcyclohexanol (MCH) (**N**). *n* = 12. (**O**) Schematic illustration of both *inc*^*1*^ and *inc*^*2*^ mutants. Note that *inc*^*2*^ is a P-element-mediated mutant with a UAS cassette for driving *inc* expression with the presence of Gal4 [31,35]. (**P**-**U**) STM (**P**), MTM (**Q**), ARM (**R**), ASM (**S**) and octanol (**T**) and MCH (**U**) odor avoidance for *inc*^*1*^. *n* =  10 for STM, 12 for MTM, 16 for ARM and ASM, and 12 for odor avoidance. (**V** and **W**) Sleep profile of *inc*^*1*^ and *inc*^*2*^ flies averaged from measurements over 2–4 days, including sleep curves plotted in 30-min bins (**V**), daily sleep amount, sleep latency at ZT12, P[doze] and P[wake] (**W**). *n* =  69–96. Except for *mns* mutants, which were in an isogenized *Canton-S* control (*Ctrl*) background, all other mutants were compared to *w*^*1118*^ (*wt*) animals. * *p* <  0.05; **p <  0.01; ***p <  0.001; ns, not significant. Error bars: mean ±  SEM. Underlying data can be found in S1 Data.

A key function of sleep is hypothesized to be the maintenance of cognitive processes [6], which is supported by evidence from the detrimental consequences of acute and chronic sleep loss, and the beneficial effects of artificially induced sleep in both humans and animal models [11,37,38]. However, the reciprocal relationship between sleep and memory formation is still not well understood on molecular, cellular and circuit levels (Fig 1A). By utilizing the well-established Pavlovian aversive olfactory conditioning [39], we initially screened large populations of a spectrum of *Drosophila* long and short sleep mutants, reasoning that chronic sleep modulations by these mutations might allow to shed light on the common molecular mechanisms of sleep alterations in regulating cognitive functions.

Interestingly, most tested mutants, regardless of whether they were long or short sleepers, displayed either normal or reduced performance compared to wild-type (*wt*)/control flies in short-term memory (STM) measured immediately after training, or middle-term memory (MTM) measured 3 h after training (Fig 1B–1L). Specifically, the short sleepers *Shaker minisleep* (*mns*) [40], 1xBRP [11,41], *homer* [42], *wide awake* (*wake*) [43] and *argus* (*aus*) [44] mutants were largely normal in olfactory associative learning and memory (Fig 1B–1L). *Neurofibromatosis-1* (*nf1*) and dopamine transporter mutant *fmn* were previously shown to regulate olfactory memory [45,46], and we could recapitulate these findings (Fig 1D, 1E and 1G). Interestingly, the long sleeping mutant *Fbxl4* [47] showed a specific decrease in STM but not MTM (Fig 1E and 1H). Notably, a single severe short sleeping mutant, *insomniac*^*2*^ (*inc*^*2*^), exhibited higher STM and MTM (Fig 1E and 1J). MTM consists of consolidated anesthesia-resistant memory (ARM) and unconsolidated anesthesia-sensitive memory (ASM), which can be distinguished by cooling trained flies on ice to induce anesthesia (also see Materials and methods) [11,21,48]. The ARM component was specifically increased in *inc*^*2*^ mutants (Fig 1K and 1L). Due to occasional unavailability of certain mutant animals during memory screening, not all memory components were tested for every mutant. Given that the *inc*^*2*^ short sleep mutants showed enhanced learning and memory, we decided to focus on dissecting the function of Inc in regulating both sleep and memory.

The *inc*^*2*^ allele is a P-element-mediated *inc* mutation with undetectable Inc protein levels in immunoblots [31,32]. To further demonstrate the important role of Inc in suppressing olfactory learning and memory, we tested the *inc*^*1*^ null mutants, which harbors a small deletion covering 5′UTR and part of the first exon of *inc* gene (Fig 1O) [31], for STM, MTM, ARM and ASM. Indeed, the *inc*^*1*^ mutants also exhibited significantly increased STM, MTM and ARM (Fig 1P–1R), confirming the essential role of Inc in constraining aversive olfactory memories. ASM was also increased in *inc*^*1*^ mutants (Fig 1S), potentially reflecting different strengths of these alleles. Both *inc*^*1*^ and *inc*^*2*^ mutants did not show any obvious alteration in odor sensation, as shown by their normal odor avoidance, proving a genuine olfactory memory phenotype in these animals (Fig 1M, 1N, 1T and 1U).

In classic Pavlovian aversive olfactory learning and memory assay, flies passively experience a conditioned stimulus (odor-electrical shock pairings), which is less dependent on internal or motivational states (e.g., foraging and mating) compared to other memory assays. Indeed, sleep has been shown to be important for a variety of distinct memory paradigms [10,37,38,41,49]. To further investigate, we examined whether *inc*^*2*^ mutants also exhibit enhanced performance in operant conditioning paradigms by studying their courtship behavior. After training naïve *wt* and *inc*^*2*^ male flies with mated *wt* females, both groups displayed substantial courtship suppression (S1A Fig). Notably, *inc*^*2*^ mutants showed significantly higher courtship learning (S1B Fig), consistent with their enhanced performance in Pavlovian aversive olfactory learning and memory (Fig 1).

Both of the two *inc* mutations were highly wake-promoting (Fig 1V, 1W and S1C–S1J Fig), consistent with previous findings [31,32]. Interestingly, *inc* mutations provoked sleep loss mainly through an increase of sleep latency and a severe decrease of sleep quality as depicted by the strongly increased probability of awaking from sleep (P[wake]), but leaving sleep pressure unaffected as indicated by the normal probability of falling asleep from wakefulness (P[doze]) [50]. In short, both *inc* alleles suffer from a substantial decrease of sleep depth and quality (Fig 1W and S1F Fig). The memory hyperfunction and sleep deficits in *inc* mutants are unlikely to result from enhanced locomotor ability, as evidenced by their decreased locomotion index (S1G–S1J Fig) and normal climbing ability (S1 Video). However, as the total daily locomotor activity was strongly increased in *inc* mutants (S1G Fig), we cannot completely exclude locomotor differences as a contributing factor to higher memory performance. Overall, these data suggest that *inc* LOF, characterized by severely decreased sleep, enhances both Pavlovian associative olfactory memory and operant courtship learning.

### Genetic modifier sleep screen of *inc* mutants

At first glance, the excessive memory in *inc* short sleep mutants seems to challenge the conventional concept that sleep loss or sleep deprivation impairs cognitive functions [51]. To better understand this apparent paradox and identify molecular mechanisms in mediating this phenotypic constellation of *inc* mutants, we undertook a modifier screen by testing autosomal heterozygous mutations for a robust modulation of the *inc* sleep phenotypes. We here focused on key players in major signaling pathways and biological processes involved in sleep regulation, circadian rhythms, learning and memory, neurotransmission and synaptic plasticity. We reasoned that any heterozygous hits identified through this screen might provide insights into the etiology of the *inc* sleep and memory phenotypes.

**Fig 2:**
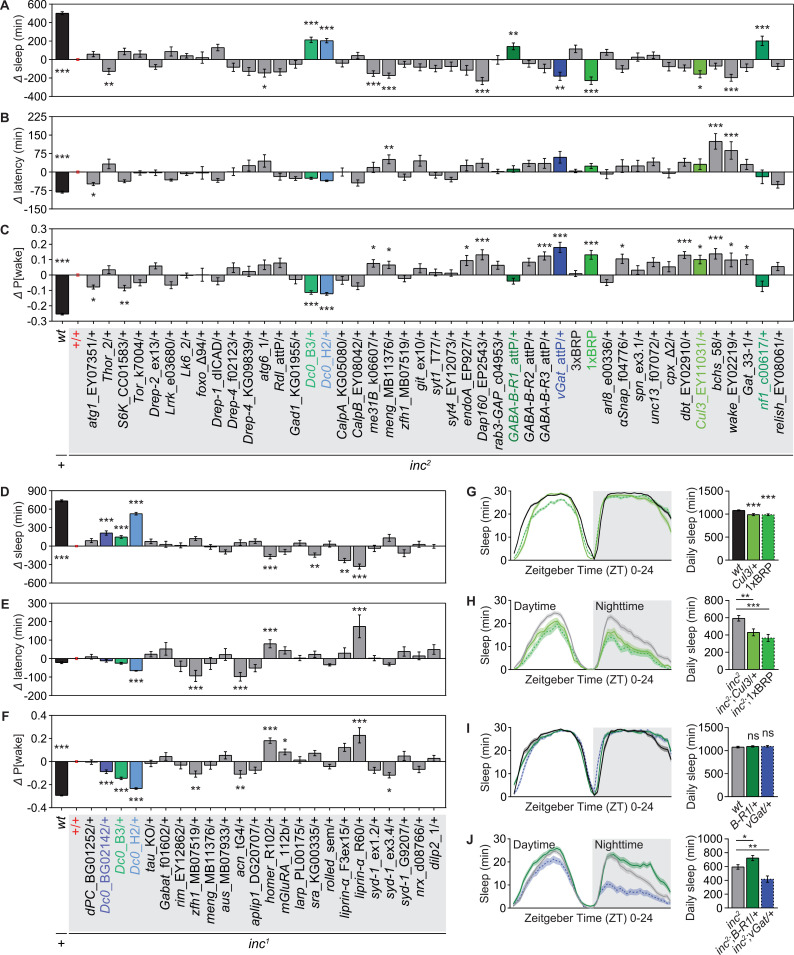
Genetic modifier sleep screen for *inc* mutants. (**A**-**C**) Genetic screen in *inc*^*2*^ mutant background analyzing the absolute difference in daily sleep (**A**), sleep latency at ZT12 (**B**) and P[wake] (**C**) between *inc*^*2*^ and *wt* or *inc*^*2*^ with autosomal heterozygous candidate mutations. *n* ≥  25. (**D**–**F**) Genetic screen in *inc*^*1*^ mutant background analyzing the absolute difference in daily sleep (**D**), sleep latency at ZT12 (**E**) and P[wake] (**F**) between *inc*^*1*^ and *wt* or *inc*^*1*^ with autosomal heterozygous candidate mutations. Note that some candidates tested in *inc*^*2*^ background were also tested in *inc*^*1*^ background in the screen. *n* = 12 for *inc*^*1*^*;liprin-α*^*R60*^/+ , 13 for *inc*^*1*^*;liprin-α*^*F3ex15*^/+ and 15 for *inc*^*1*^*;syd-1*^*ex3.4*^/+ , *n* ≥ 23 for all other genotypes. (**G**–**J**) Some examples of candidates shown to tune sleep of *inc* mutants by their heterozygosity, including *Cullin-3*^*EY11031*^/+ (*Cul3*/+) and *brp*^*c04298*^/+ (1xBRP) (**G** and **H**), and *GABA-B-R1*^*attP*^/+ (*B-R1*/+) and *vGat*^*attP*^/+ (*vGat*/+) (**I** and **J**). *n* = 40–111 for **G**, 30–51 for **H**, 40–47 for **I**, and 30–51 for **J**. Statistical comparisons were performed either between *wt* and *inc*, or between *inc* with or without heterozygous candidate mutations. *p < 0.05; **p < 0.01; ***p < 0.001; nonsignificant comparisons are not shown for simplicity. Error bars: mean ±  SEM. Underlying data can be found in S2 Data.

Loss of *inc* suppresses sleep mainly by promoting nighttime sleep latency and decreasing sleep quality indicated by higher P[wake] (Fig 1W). Thus, in our modifier sleep screen, we quantified daily sleep amount, nighttime sleep latency and P[wake], and calculated the absolute differences between *inc* mutant flies with or without the presence of heterozygous candidate mutations (Fig 2A–2F). We screened ~70 isogenized alleles, finding that *inc* mutants were sensitive for heterozygous modifiers further exaggerating their short sleep phenotypes (Fig 2A and 2D). As we showed previously [41], removing a single copy of the ELKS-family active zone scaffold protein Bruchpilot (1xBRP) was more profound in promoting wakefulness in *inc* mutants than in *wt*/control background (Fig 2G, 2H and S2A, S2B Fig). As mentioned above already, Inc functions as an adaptor of the E3 ligase Cullin-3 [31]. Consistent with the role of Inc in promoting Cullin-3 function to regulate sleep, *Cullin-3* heterozygosity further enhanced the *inc* sleep deficits (Fig 2G, 2H and S2A, S2B Fig). Moreover, genetic modulations of GABAergic signaling dynamically modified the *inc* sleep phenotypes (Fig 2I, 2J and S2C, S2D Fig).

In contrast to these potential hits enhancing the *inc* short sleep phenotypes, only a few modifiers were able to rescue the sleep phenotypes of *inc* mutants. The strongest and most robust rescue was observed after establishing heterozygosity for the PKA catalytic subunit Dc0, particularly pronounced for the *Dc0*^*H2*^ allele in *inc*^*1*^ background (Fig 2A–2F). Moreover, Neurofibromatosis-1 (Nf1), previously shown to regulate cAMP/PKA signaling in the context of sleep and memory regulation [45,52], also significantly suppressed the short sleep phenotypes of *inc* mutants by its heterozygosity (Fig 2A–2C). Given the consistent rescue effects after a mild genetic reduction of PKA signaling components, we decided to focus our efforts on the potential relationship between Inc and the cAMP/PKA signaling pathway.

### cAMP/PKA signaling mediates the sleep phenotypes of *inc* mutants

**Fig 3:**
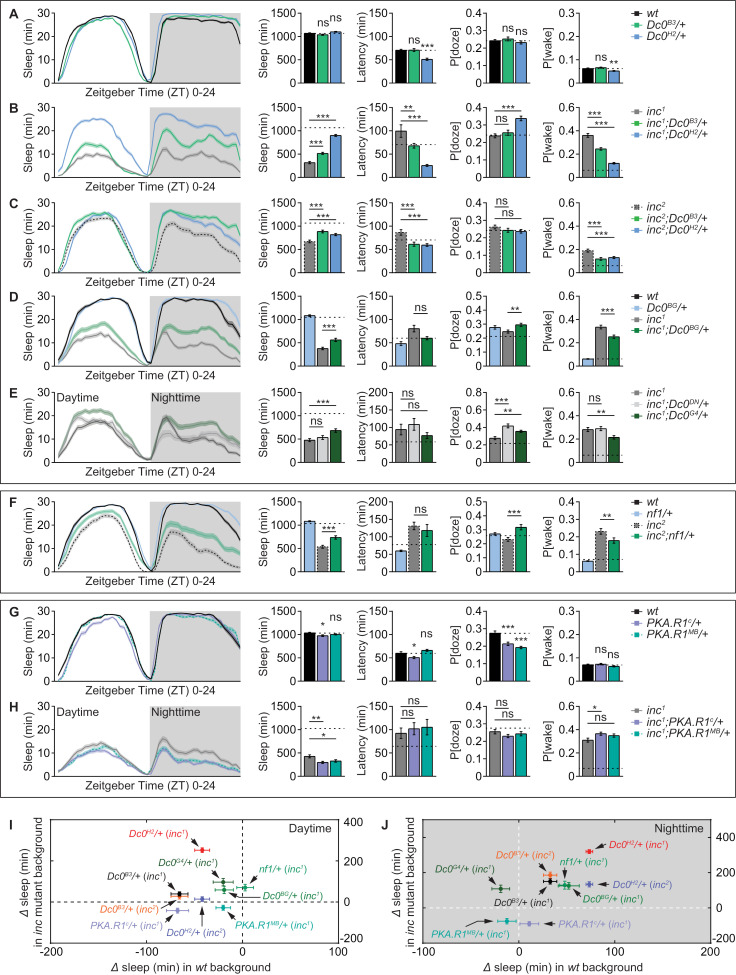
cAMP/PKA signaling mediates the sleep phenotypes of *inc* mutants. (**A**) Sleep profile of *wt*, *Dc0*^*B3*^/+ and *Dc0*^*H2*^/+ flies averaged from measurements over 2–4 days, including sleep curves plotted in 30-min bins, daily sleep amount, sleep latency at ZT12, P[doze] and P[wake]. *n* = 70–76. (**B**) Sleep profile of *inc*^*1*^ with either *Dc0*^*B3*^/+ or *Dc0*^*H2*^/+ averaged from measurements over 2–4 days, including sleep curves plotted in 30-min bins, daily sleep amount, sleep latency at ZT12, P[doze] and P[wake]. *n* = 57–66. (**C**) Sleep profile of *inc*^*2*^ with either *Dc0*^*B3*^/+ or *Dc0*^*H2*^/+ averaged from measurements over 2–4 days, including sleep curves plotted in 30-min bins, daily sleep amount, sleep latency at ZT12, P[doze] and P[wake]. *n* = 73–76. (**D**) Sleep profile of *wt*, *Dc0*^*BG*^/+ and *inc*^*1*^ with or without *Dc0*^*BG*^/+ averaged from measurements over 2–4 days, including sleep curves plotted in 30-min bins, daily sleep amount, sleep latency at ZT12, P[doze] and P[wake]. *n* = 40–71. (**E**) Sleep profile of *wt* and *inc*^*1*^ with either *Dc0*^*DN*^/+ or *Dc0*^*G4*^/+ averaged from measurements over 2–4 days, including sleep curves plotted in 30-min bins, daily sleep amount, sleep latency at ZT12, P[doze] and P[wake]. *n* = 32–48. (**F**) Sleep profile of *wt*, *nf1*/+ and *inc*^*2*^ with or without *nf1*/+ averaged from measurements over 2–4 days, including sleep curves plotted in 30-min bins, daily sleep amount, sleep latency at ZT12, P[doze] and P[wake]. *n* = 62–80. (**G**) Sleep profile of *wt*, *PKA.R1*^*c*^/+ and *PKA.R1*^*MB*^/+ flies averaged from measurements over 4 days, including sleep curves plotted in 30-min bins, daily sleep amount, sleep latency at ZT12, P[doze] and P[wake]. *n* = 58–63. (**H**) Sleep profile of *inc*^*1*^ with either *PKA.R1*^*c*^/+ or *PKA.R1*^*MB*^/+ averaged from measurements over 4 days, including sleep curves plotted in 30-min bins, daily sleep amount, sleep latency at ZT12, P[doze] and P[wake]. *n* = 61–67. Dashed straight lines indicate mean values of *wt*/control animals tested simultaneously. *p < 0.05; **p < 0.01; ***p < 0.001; ns, not significant. Error bars: mean ± SEM. (**I** and **J**) Absolute differences in daytime (**I**) and nighttime (**J**) sleep among different heterozygotes of cAMP/PKA signaling components in *wt* or *inc* mutant backgrounds in **A**–**H**. *p < 0.05; **p < 0.01; ***p < 0.001; ns, not significant. Error bars: mean ±  SEM. Underlying data can be found in S3 Data.

To deepen our understanding towards the exact nature of this rescue scenario, we examined the detailed sleep phenotypes of the two *inc* mutants in conjunction with heterozygosity of several distinct alleles of the PKA catalytic subunit *Dc0* (Fig 3A–3E). *Dc0*^*B3*^ and *Dc0*^*H2*^ alleles are LOF mutants harboring point mutations within the *Dc0* open reading frame [53]. Heterozygosity of *Dc0*^*B3*^ and *Dc0*^*H2*^ was previously shown to trigger moderate but significant reductions in PKA activity [29]. We first found that, while *Dc0*^*H2*^ heterozygosity provoked more consolidated and deeper sleep than *Dc0*^*B3*^ heterozygosity in *wt*/control background indicated by shorter sleep latency and lower P[wake], both alleles exhibited mild alterations in sleep pattern with a slight reduction in daytime sleep and an increase in nighttime sleep, and an overall normal daily sleep amount (Fig 3A and S3A Fig). These data indicate that, different from strong manipulations in cAMP/PKA signaling [28], a mild reduction in PKA signaling triggered by *Dc0* heterozygosity has no major sleep phenotypes in *wt*/control background.

Notably, both daytime and nighttime sleep were substantially restored for both *inc*^*1*^ and *inc*^*2*^ mutants by heterozygosity of either *Dc0*^*B3*^ or *Dc0*^*H2*^ compared to *inc*^*1*^ or *inc*^*2*^ mutants, with shortened sleep latency and severely reduced P[wake], indicating higher sleep quality (Fig 3B, 3C and S3B, S3C Fig). As noted earlier (Fig 2D), the sleep restoring effects of *Dc0*^*H2*^ allele were much stronger in *inc*^*1*^ than in *inc*^*2*^ background, suggesting a higher sleep pressure provoked by this allele (Fig 3B and S3B Fig). Indeed, *Dc0*^*H2*^ heterozygosity was shown to have lower PKA activity when compared to *Dc0*^*B3*^ heterozygosity [29]. Thus, the *Dc0* allele-specific sleep effects in *inc* mutant backgrounds might reflect the allelic strength of these PKA alleles (Fig 3I and 3J).

To further explore the role of PKA signaling in regulating the sleep phenotypes of *inc* mutants and to particularly address whether these effects were *Dc0* allele-specific, we also utilized two P-element-mediated mutations (*Dc0*^*BG*^ and *Dc0*^*G4*^ lines) and one additional dominant negative mutant (*Dc0*^*DN*^ line). These three additional lines are all adult homozygous lethal, but only *Dc0*^*BG*^ and *Dc0*^*G4*^ in heterozygosity showed similar effects on restoring sleep to *inc* mutants when compared to *Dc0*^*B3*^ (Fig 3D, 3E and S3D, S3E Fig), while *Dc0*^*DN*^ as a dominant negative allele distinct from the *Dc0* null mutants [54] had no effects on the sleep of *inc* mutants (Fig 3E and S3E Fig). Our data thus show that heterozygosity of *Dc0* null or strong hypomorphic alleles consistently suppresses the sleep phenotypes of *inc* mutants, while having mild to no effects in *wt*/control animals (Fig 3I, 3J and S3I–S3L Fig). Thus, it seems that *inc* mutants are sensitized towards PKA signaling modulation, and the level of PKA signaling seems to be directly associated with the severity of their sleep deficits (Fig 3I and 3J).

In its inactive state, PKA forms a tetramer consisting of two catalytic and two regulatory subunits [55]. cAMP activates PKA by binding to the regulatory subunits and subsequently releasing the catalytic subunits so as to phosphorylate target proteins [55]. Thus, the regulatory subunit directly antagonizes the kinase function of the catalytic subunit, and the synthesis of cAMP by adenylate cyclases such as Rutabaga (Rut) is essential for activating/disinhibiting PKA signaling. Along this line, in addition to Dc0, modulating other regulatory components of cAMP/PKA signaling could potentially also tune the sleep phenotypes of *inc* mutants.

Through our genetic modifier sleep screen for *inc* mutants (Fig 2), we identified Nf1 as a significant modifier (Fig 2A–2C). *nf1* heterozygosity restored a great extent of sleep in *inc* mutant background by increasing P[doze] and simultaneously reducing P[wake], whereas it had only very mild effects in *wt*/control background (Fig 3F, 3I, 3J and S3F, S3M Fig). In *Drosophila*, Nf1 has been studied in the regulation of circadian rhythms [56], sleep [52,57,58] and memory (Fig 1D and 1G) [45]. Importantly, Nf1 was shown to be proteasome-degraded through Cullin-3 E3 ligase-mediated ubiquitination *in vitro* [59]. Furthermore, Nf1 promotes cAMP/PKA signaling by increasing Rut adenylate cyclase activity and subsequently the synthesis of cAMP and activation of PKA signaling [45]. Thus, it seems possible that a hierarchical signaling cascade, normally elicited by Inc/Cullin-3 complex-mediated Nf1 degradation and subsequent PKA activity suppression, feeds forward to fine-tune the levels of sleep (see Discussion and S6D Fig).

To potentially tune PKA signaling bidirectionally, we further tried to increase PKA signaling by establishing heterozygous scenarios for PKA regulatory subunit type 1 (PKA.R1) both in *wt*/control and *inc* backgrounds. In contrast to *Dc0* heterozygosity (Fig 3A–3E and S3A–S3E Fig), the heterozygosity of two alleles of *PKA.R1* enhanced the short sleep phenotypes of *inc* mutants, while having very mild or no obvious effect in *wt*/control background (Fig 3G–3J and S3G, S3H, S3N, S3O Fig). These data again suggest that *inc* mutants are sensitized towards bidirectional changes of PKA signaling, which seems to directly mediate the sleep phenotypes of *inc* mutants.

### The effects of *Dc0* heterozygosity on promoting sleep are specific to *inc* mutants

**Fig 4:**
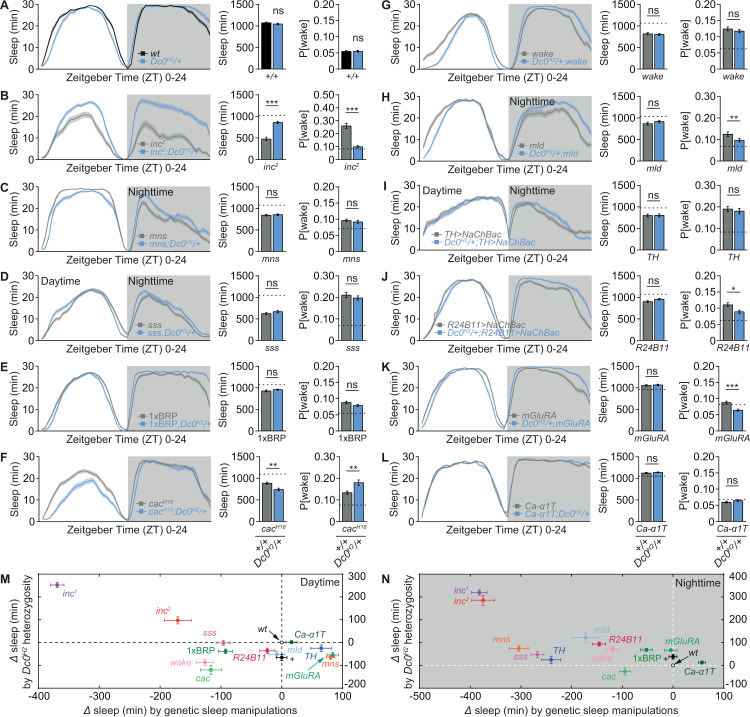
The effects of *Dc0* heterozygosity on promoting sleep are specific to *inc* mutants. (**A**-**L**) Sleep profile of *Dc0*^*H2*^/+ in control (**A**), *inc*^*2*^ (**B**), *mns* (**C**), *sss* (**D**), 1xBRP (**E**), *cac*^*H18*^ (**F**), *wake* (**G**), *mld* (**H**), *TH>NaChBa*c (**I**), *R24B11 > NaChBac* (**J**), *mGluRA* (**K**) and *Ca-*α*1T* (**L**) backgrounds, averaged from measurements over 2–4 days, including sleep curves plotted in 30-min bins, daily sleep amount, and P[wake]. *n* = 48–52 for **A**, 32–38 for **B**, 48–56 for **C**, 47–72 for **D**, 48–51 for **E**, 48 for **F**, 54–56 for **G**, 29–48 for **H**, 32–48 for **I** and **J**, 39–47 for **K**, and 48 for **L**. Dashed lines indicate mean values of *wt*/control animals tested simultaneously. (**M** and **N**) Absolute differences in daytime (**M**) and nighttime (**N**) sleep among different genetic sleep manipulations in **A**–**L** and the diverse effects of *Dc0*^*H2*^/+ in these backgrounds of manipulations. *p < 0.05; **p < 0.01; ***p < 0.001; ns, not significant. Error bars: mean ±  SEM. Underlying data can be found in S4 Data.

The PKA kinase targets a spectrum of downstream effectors and has widespread effects on various biological processes [60, 61]. We thus asked whether the effects of *Dc0*^*H2*^ heterozygosity on sleep are specific for *inc* mutants (Figs 3A–3C, 4A and 4B), or generalize to other genetic sleep deficit scenarios as well. For this purpose, we chose two different approaches: (1) distinct short and long sleep mutants (Fig 4C–4H, 4K and 4L) and (2) activating previously described wake-promoting sleep circuits (Fig 4I and 4J). For most of these scenarios, we did not observe any interference by *Dc0*^*H2*^ heterozygosity on daily sleep, including the short-sleeping voltage-gated potassium channel *Shaker mns* mutants [40] (Fig 4C and S4C Fig), *sleepless* (*sss*) mutants [62] (Fig 4D and S4D Fig), 1xBRP [11,41] (Fig 4E and S4E Fig), *wide awake* (*wake*) mutants [43] (Fig 4G and S4G Fig), *molting defective* (*mld*) mutants [63] (Fig 4H and S4H Fig), and long-sleeping *mGluRA* mutants [64] (Fig 4K and S4K Fig) and *T-type like voltage-gated calcium channel* (*Ca-α1T*) mutants [65] (Fig 4L and S4L Fig). Interestingly, the short sleep phenotype of *cacophony*^*H18*^ (*cac*^*H18*^) [66] was further enhanced (Fig 4F and S4F Fig). Furthermore, the *Dc0*^*H2*^ heterozygous scenario did not interfere with the reduced daily sleep caused by the activation of dopaminergic neurons [67] or helicon cells [68] by expressing low-threshold voltage-gated sodium channel NaChBac [69] (Fig 4I, 4J and S4I, S4J Fig). Taken together, our data show that *inc* mutants are specifically sensitized towards a reduction of PKA signaling.

As mentioned earlier, it is worth noting that *Dc0*^*H2*^ heterozygosity drove a slight re-organization of daytime and nighttime sleep, indicated by a slight decrease of sleep during daytime but a mild increase of sleep during nighttime, resulting in an indistinguishable total daily sleep amount compared to *wt* (Figs 3A and 4A). Consistently, cAMP/PKA signaling was previously shown to regulate circadian rhythms [70], and *Dc0* expression exhibits daily oscillations [52], suggesting different functions for the regulation of daytime versus nighttime sleep. We next systematically compared the effects of *Dc0*^*H2*^ heterozygosity in different backgrounds of genetic sleep manipulations, taking daytime and nighttime into separate considerations (Fig 4M and 4N). Indeed, *Dc0*^*H2*^ heterozygosity was wake-promoting during daytime in most cases, except for *inc* mutants, in which it became sleep-promoting (Fig 4M). During nighttime, *Dc0*^*H2*^ heterozygosity generally showed rather minor sleep-promoting effects, which became much more prominent in *inc* mutant background (Fig 4N).

### Mushroom body PKA kinase activity is increased in *inc* mutants

**Fig 5:**
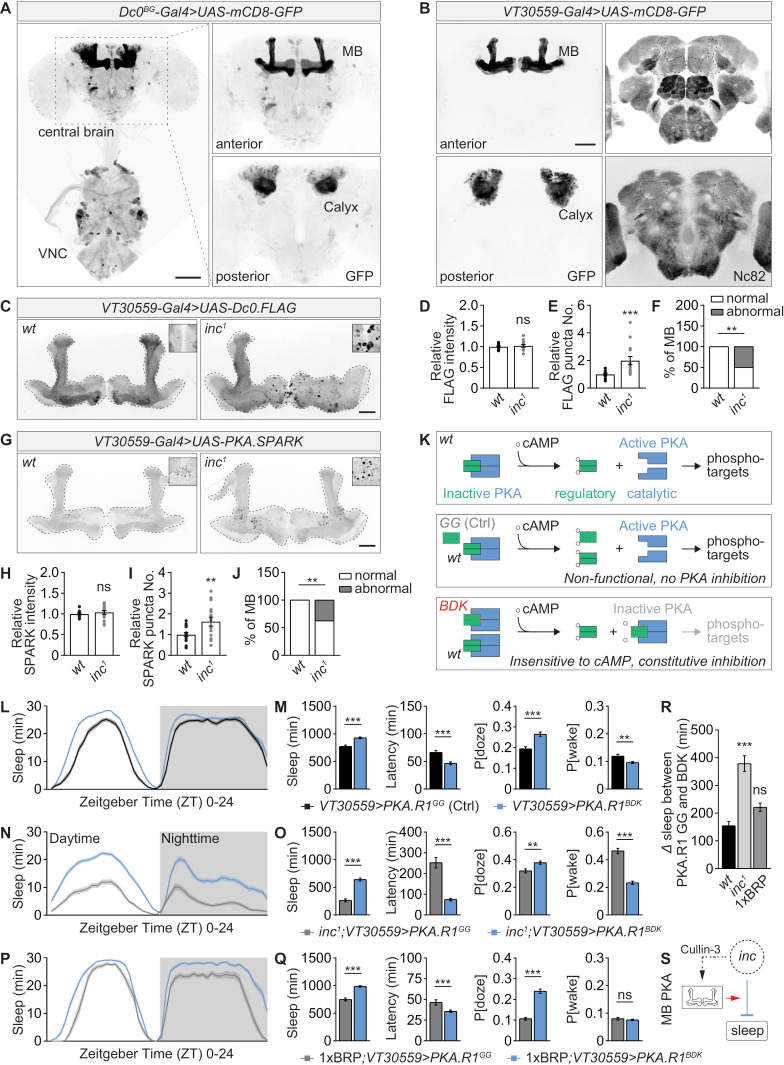
Elevated PKA kinase activity in the mushroom body drives sleep deficits of *inc* mutants. (**A**) Whole-mount brain expressing mCD8-GFP driven by the *Dc0*^*BG*^ Gal4 gene trap line demonstrating the expression pattern of Dc0 in brain and ventral nerve cord (VNC). Dc0 has broad expression but highly enriched in the mushroom body (MB) lobes and calyx. Native GFP fluorescence is shown. Scale bar: 100 μm. (**B**) Whole-mount brain expressing mCD8-GFP driven the transgenic *VT30559-Gal4* line demonstrating its highly restricted MB-specific expression pattern. The brains were stained with Nc82 antibody, and native GFP fluorescence is shown. Scale bar: 50 μm. (**C**–**F**) Whole-mount brain immunostaining against FLAG for *wt*/control and *inc*^*1*^ flies expressing Dc0-FLAG in the mushroom body driven by *VT30559-Gal4*, including representative images (**C**), average intensity (**D**) and FLAG puncta number (**E**). *inc* mutants exhibited abnormal mushroom body lobe morphology (**F**). *n* = 14. Scale bar: 20 μm. (**G**–**J**) Whole-mount brain immunostaining against GFP for *wt*/control and *inc*^*1*^ flies expressing PKA kinase activity sensor PKA.SPARK in the mushroom body driven by *VT30559-Gal4*, including representative images (**G**), average intensity (**H**) and SPARK puncta number (**I**). *inc* mutants again exhibited abnormal mushroom body lobe morphology (**J**). *n* = 15−16. Scale bar: 20 μm. (**K**) Simplified schematic illustration for the regulation of PKA kinase activity by cAMP and PKA regulatory subunit in *wt* background or under the expression of either a non-functional regulatory subunit (PKA.R1^GG^), which does no longer bind to the catalytic subunit, or a constitutive active regulatory subunit (PKA.R1^BDK^), which constitutively binds to the catalytic subunit and is insensitive to cAMP. (**L** and **M**) Sleep profile from measurements over 4 days of flies expressing non-functional (GG) or active (BDK) PKA regulatory subunit in the mushroom body driven by *VT30559-Gal4* in *wt*/control background, including sleep curves plotted in 30-min bins (**L**), daily sleep amount, sleep latency at ZT12, P[doze] and P[wake] (**M**). *n*= 76. (**N** and **O**) Sleep profile from measurements over 4 days of flies expressing non-functional (GG) or active (BDK) PKA regulatory subunit PKA.R1 in the mushroom body driven by *VT30559-Gal4* in *inc*^*1*^ mutant background, including sleep curves plotted in 30-min bins (**N**), daily sleep amount, sleep latency at ZT12, P[doze] and P[wake] (**O**). *n* = 83−85. (**P** and **Q**) Sleep profile from measurements over 2−4 days of flies expressing non-functional (GG) or active (BDK) PKA regulatory subunit PKA.R1 in the mushroom body driven by *VT30559-Gal4* in 1xBRP background, including sleep curves plotted in 30-min bins (**P**), daily sleep amount, sleep latency at ZT12, P[doze] and P[wake] (**Q**). *n* = 27−48. (**R**) Absolute difference in daily sleep between flies expressing non-functional (GG) and active (BDK) PKA regulatory subunit in the mushroom body in *wt*/control, *inc*^*1*^ or 1xBRP background. *n* = 48−83. (**S**) Simplified model suggesting that the Inc/Cullin-3 complex regulates mushroom body PKA signaling to promote sleep. **p < 0.01; ***p   0.001; ns, not significant. Error bars: mean ±  SEM. Underlying data can be found in S5 Data.

Inc was reported to be broadly expressed in the nervous system [31,32], including the mushroom body within which Inc was shown to contribute to sleep-promoting effects [35]. To directly visualize the expression pattern of Dc0 by driving GFP reporter expression, we utilized the *Dc0*^*BG*^ allele, which is a *Gal4* gene trap of the *Dc0* locus and whose heterozygosity (*Dc0*^*BG*^/+) rescued the sleep of *inc* mutants (Fig 3D). We observed that Dc0 was also broadly expressed in adult central brain and ventral nerve cord, with particularly prominent expression in the mushroom body (Fig 5A), consistent with previous reports [29,71]. Given that a mild reduction in PKA signaling in *Dc0*/+ rescued the sleep phenotypes of *inc* mutants, we asked whether the Dc0 protein level was directly changed in *inc* mutants. Due to the lack of a functional antibody targeting Dc0, we expressed and visualized FLAG-tagged Dc0 in the mushroom body Kenyon cells driven by the mushroom body-specific *VT30559-Gal4* (Fig 5B). Importantly, Gal4-driven expressed Dc0-FLAG localized to the mushroom body properly in *wt* background (Fig 5C), similar to Dc0 antibody staining [29]. The overall Dc0-FLAG staining intensity did not differ between *wt* and *inc* mutants (Fig 5C and 5D), suggesting that Dc0 protein abundance is unlikely to be regulated by Inc. However, the distribution of Dc0-FLAG was markedly altered in *inc* mutants, characterized by circular structures with highly concentrated Dc0-FLAG staining (Fig 5C and 5E). This pattern may indicate altered PKA kinase function and signaling. Additionally, *inc* mutants displayed abnormal mushroom body morphology, including overgrown structures and missing lobes (Fig 5C and 5F), consistent with previous findings [35,71].

To directly assess the PKA kinase activity level, we expressed the PKA activity reporter PKA.SPARK [72] in the mushroom body Kenyon cells driven by *VT30559-Gal4*. Upon PKA activation, SPARK signal forms puncta from otherwise diffuse signal *in vitro* and *in vivo* [72,73]. Consistent with this, we observed an increase in the number of PKA SPARK puncta in *inc* mutants, while the average level of SPARK intensity did not change (Fig 5G–5I). Additionally, structural abnormalities in the mushroom body were evident in *inc* mutants (Fig 5G and 5J). These data suggest elevated PKA kinase activity in *inc* mutant Kenyon cells, which might be directly causal for the severe sleep defects of *inc* mutants.

As mentioned above, PKA forms a tetramer consisting of two catalytic and two regulatory subunits [55] (Fig 5K). In its inactive state, PKA catalytic subunit kinase activity is suppressed by its regulatory subunit [55] (Fig 5K). cAMP then activates PKA by binding to the regulatory subunits and subsequently releases and disinhibits the catalytic subunits so as to allow for phosphorylating target proteins [55] (Fig 5K). As shown earlier, reducing the level of PKA regulatory subunit could further reduce the sleep of *inc* mutants (Fig 3H). To further test whether PKA kinase activity within the mushroom body is causal for the sleep deficits of *inc* mutants, we expressed an active form of the regulatory subunit (PKA.R1^BDK^), which is insensitive to cAMP and constitutively inhibits the catalytic subunit (Fig 5K) [74], to suppress PKA kinase activity in the mushroom body. As control, a non-functional form (PKA.R1^GG^), which has no binding activity to the catalytic subunit, was expressed in the mushroom body. We first show that expressing PKA.R1^GG^ in the mushroom body driven by *VT30559-Gal4* did not significantly affect sleep in both *wt* and *inc* mutant backgrounds (S5A and S5B Fig). Interestingly, we found that expression of PKA.R1^BDK^ triggered a moderate increase of sleep when compared to PKA.R1^GG^ expression in *wt*/control background (Fig 5L, 5M and S5C Fig). The sleep-promoting effects of mushroom body PKA.R1^BDK^ expression were more pronounced in *inc* mutant background, but not in 1xBRP background (Fig 5N–5R and S5D, S5E Fig), similar to the effects of *Dc0*^*H2*^ heterozygosity (Figs 3B, 3C and 4B). Collectively, these data suggest that a higher PKA kinase activity within the mushroom body contributes to the *inc* sleep phenotypes.

### Elevated PKA signaling contributes to the compromised life expectancy of *inc* mutants

**Fig 6:**
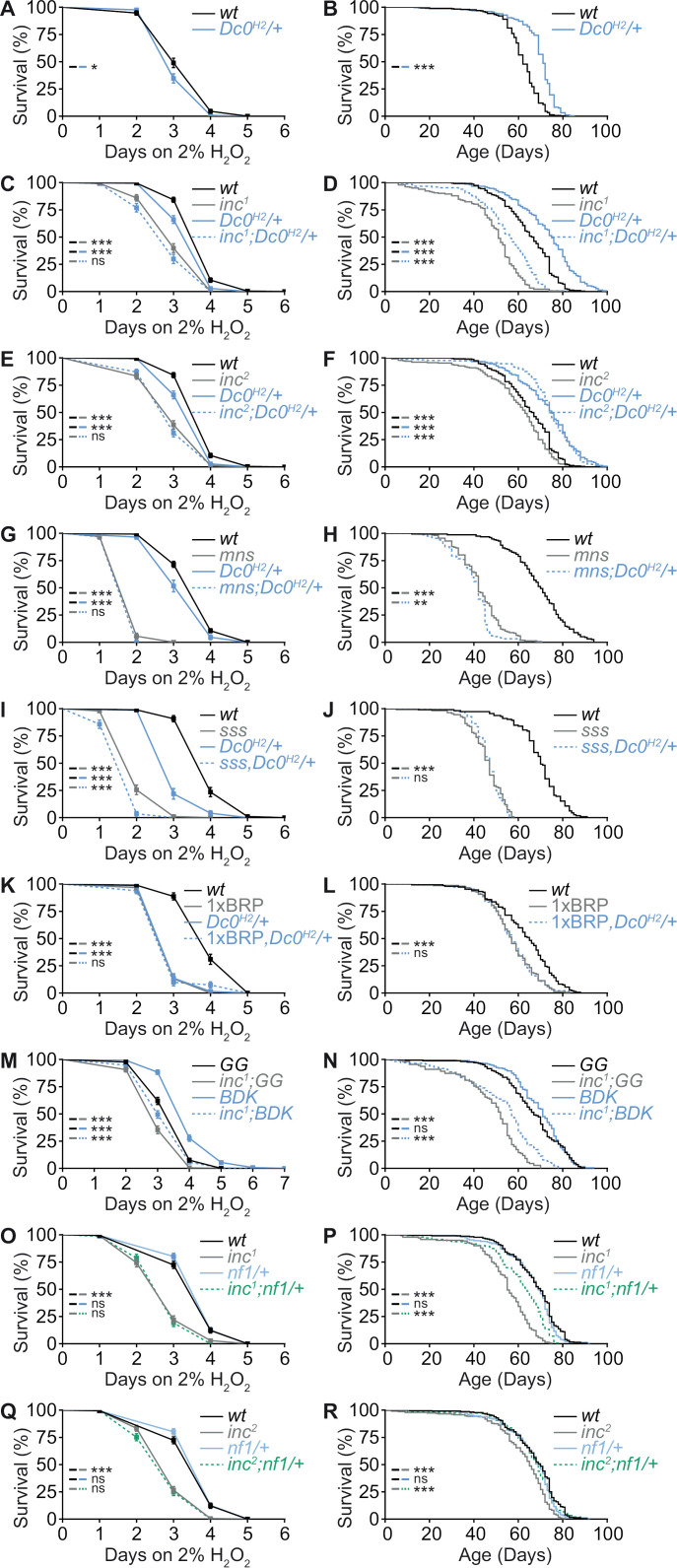
cAMP/PKA signaling mediates the longevity phenotypes of *inc* mutants. (**A** and **B**) Survival curves under 2% H_2_O_2_ treatment (**A**) or normal aging (**B**) for *wt* and *Dc0*^*H2*^/+ flies. For H_2_O_2_ treatment, *n* = 133 for *wt* and 124 for *Dc0*^*H2*^/+. For longevity, *n* = 299 for *wt* and 280 for *Dc0*^*H2*^/+. (**C** and **D**) Survival curves under 2% H_2_O_2_ treatment (**C**) or normal aging (**D**) for *wt*, *Dc0*^*H2*^/+ and *inc*^*1*^ with or without *Dc0*^*H2*^/+. (**E** and **F**) Survival curves under 2% H_2_O_2_ treatment (**E**) or normal aging (**F**) for *wt*, *Dc0*^*H2*^/+ and *inc*^*2*^ with or without *Dc0*^*H2*^/+. Note that **C** and **E** share the same *wt* and *Dc0*^*H2*^/+, as well as in **D** and **F**. For H_2_O_2_ treatment, *n* = 228 for *wt*, 177 for *Dc0*^*H2*^/+, 143 for *inc*^*1*^, 163 for *inc*^*2*^, 117 for *inc*^*1*^;*Dc0*^*H2*^/+ and 170 for *inc*^*2*^;*Dc0*^*H2*^/+. For longevity, *n* = 234 for *wt*, 150 for *Dc0*^*H2*^/+, 182 for *inc*^*1*^, 232 for *inc*^*2*^, 149 for *inc*^*1*^;*Dc0*^*H2*^/+ and 225 for *inc*^*2*^;*Dc0*^*H2*^/+. (**G** and **H**) Survival curves under 2% H_2_O_2_ treatment (**G**) or normal aging (**H**) for *wt*, *Dc0*^*H2*^/+ and *mns* with or without *Dc0*^*H2*^/+. For H_2_O_2_ treatment, *n* = 264 for *wt*, 87 for *Dc0*^*H2*^/+, 71 for *mns* and 62 for *mns*;*Dc0*^*H2*^/+. For longevity, *n* = 156 for *wt*, 156 for *mns* and 154 for *mns*;*Dc0*^*H2*^/+. (**I** and **J**) Survival curves under 2% H_2_O_2_ treatment (**I**) or normal aging (**J**) for *wt*, *Dc0*^*H2*^/+ and *sss* with or without *Dc0*^*H2*^/+. For H_2_O_2_ treatment, *n* = 98 for *wt*, 73 for *Dc0*^*H2*^/+, 98 for *sss* (*n* = 98, *p* <  0.001) and 84 for *sss*,*Dc0*^*H2*^/+. For longevity, *n* = 112 for *wt*, 110 for *sss* and 109 for *sss*,*Dc0*^*H2*^/+. (**K** and **L**) Survival curves under 2% H_2_O_2_ treatment (**K**) or normal aging (**L**) for *wt*, *Dc0*^*H2*^/+ and 1xBRP with or without *Dc0*^*H2*^/+. For H_2_O_2_ treatment, *n* = 97 for *wt*, 66 for *Dc0*^*H2*^/+, 91 for 1xBRP and 81 for 1xBRP,*Dc0*^*H2*^/+. For longevity, *n* = 147 *wt*, 150 for 1xBRP and 139 for 1xBRP,*Dc0*^*H2*^/+. (**M** and **N**) Survival curves under 2% H_2_O_2_ treatment (**M**) or normal aging (**N**) for flies expressing non-functional (GG) or active (BDK) PKA regulatory subunit PKA.R1 in the mushroom body driven by *VT30559-Gal4* in *wt* and *inc*^*1*^ mutant backgrounds. For H_2_O_2_ treatment, *n* = 210 for *GG*, 238 for *BDK*, 181 for *inc*^*1*^*;GG* and 210 for *inc*^*1*^;*BDK*. For longevity, *n* = 165 for *GG*, 189 for *BDK*, 110 for *inc*^*1*^*;GG* and 168 for *inc*^*1*^;*BDK*. (**O** and **P**) Survival curves under 2% H_2_O_2_ treatment (**O**) or normal aging (**P**) for *wt*, *nf1*/+ and *inc*^*1*^ with or without *nf1*/+. (**Q** and **R**) Survival curves under 2% H_2_O_2_ treatment (**Q**) or normal aging (**R**) for *wt*, *nf1*/+ and *inc*^*2*^ with or without *nf1*/+. Note that **O** and **Q** share the same *wt* and *nf1*/+, as well as in **P** and **R**. For H_2_O_2_ treatment, *n* = 188 for *wt*, 191 for *nf1*/+, 136 for *inc*^*1*^, 180 for *inc*^*2*^, 141 for *inc*^*1*^;*nf1*/+, and 177 for *inc*^*2*^;*nf1*/+. For longevity, *n* = 227 for *wt*, 148 for *nf1*/+, 140 for *inc*^*1*^, 232 for *inc*^*2*^, 143 for *inc*^*1*^;*nf1*/+ (*n* = 143) and 148 for *inc*^*2*^;*nf1*/+ (*n* = 148). *p < 0.05; **p < 0.01; ***p < 0.001; ns, not significant. Error bars: ±  standard error (SE). Underlying data can be found in S6 Data.

Similar to other short-sleep mutants, *inc* animals were previously shown to be short-lived [31] and hypersensitive to oxidative stress [13]. Given the strong rescue effects of *Dc0* heterozygosity toward the *inc* mutant sleep deficits, we hypothesized that it might also rescue their survival under oxidative stress and longevity phenotypes. For this purpose, we first measured the survival of the *Dc0*^*H2*^ heterozygous animals treated with 2% hydrogen peroxide (H_2_O_2_) and their life span upon normal aging. Interestingly, *Dc0*^*H2*^ heterozygous animals were more vulnerable than *wt*/control in response to 2% H_2_O_2_ treatment (Fig 6A). In *wt*/control background, the life span of *Dc0*^*H2*^ heterozygous animals was significantly increased compared to *wt*/control (Fig 6B), consistent with a previously reported tendency of increased life span in *Dc0*^*H2*^ heterozygous flies [29]. We then tested *Dc0*^*H2*^ heterozygosity in both *inc*^*1*^ and *inc*^*2*^ mutants. Similar to a previous report [13], we confirmed that *inc* mutants were hypersensitive to H_2_O_2_ treatment, while *Dc0*^*H2*^ heterozygosity did not have any additive effect here (Fig 6C and 6E). However, in terms of longevity, *inc*^*1*^ mutants were clearly improved and *inc*^*2*^ mutants were fully rescued to the extent of *Dc0*^*H2*^ heterozygous animals (Fig 6D and 6F). These improvements in longevity, accompanied by a significant rescue in sleep (Fig 3B and 3D), suggest a healthier physiological state being established for *inc* mutants by a mild down-regulation of PKA signaling. Again, to test the specificity of this rescue of longevity in the absence of a rescue in the survival upon H_2_O_2_ treatment, we wondered if *Dc0*^*H2*^ heterozygosity was also sufficient to improve the survival of other short-sleeping scenarios including *Shaker mns*, *sss* and 1xBRP upon H_2_O_2_ treatment and in longevity. Notably, however, for these short sleep mutants, no rescue was observed (Fig 6G–6L). Thus, our data suggest the scenario that a moderate downregulation of PKA signaling is sufficient to specifically promote longevity as a trait unique to *inc* among the tested short sleep mutants.

Given that suppressing PKA signaling by PKA.R1^BDK^ expression specifically in the mushroom body was sufficient to restore sleep in *inc* mutants (Fig 5L–5R), we next asked if longevity and survival upon H_2_O_2_ treatment could also be rescued. Consistent with the effects of *Dc0* heterozygosity on longevity (Fig 6B, 6D and 6F), mushroom body-specific PKA.R1^BDK^ expression showed a clear tendency towards extended life span in *wt* background and significantly increased the life span of *inc* mutants (Fig 6N). Interestingly, these flies were also longer-lived upon H_2_O_2_ treatment when compared to control flies expressing PKA.R1^GG^ in both *wt* and *inc* mutant backgrounds (Fig 6M), different from the effects of *Dc0* heterozygosity (Fig 6A, 6C and 6E). These data suggest that mushroom body-specific PKA suppression promotes survival during both normal aging and acute oxidative stress.

To test if the rescue effects on longevity of *inc* mutants was specific to *Dc0* heterozygosity or would extend to other PKA signaling components, we further measured the longevity as well as survival upon H_2_O_2_ treatment of *nf1* heterozygosity in *wt*/control and *inc* mutant backgrounds, which was shown above to restore sleep to *inc* mutants (Fig 3F). While *nf1* heterozygosity did not show any obvious difference in both survival paradigms compared to *wt*/control (Fig 6O–6R), it substantially improved the longevity of *inc* mutants (Fig 6P and 6R), leaving their survival phenotype upon H_2_O_2_ treatment unaffected (Fig 6O and 6Q). Thus, it is likely that a mild reduction in PKA signaling, provoked by the heterozygosity of PKA regulatory component *nf1*, triggered effects on the longevity of *inc* mutants comparable to those observed with *Dc0* heterozygosity. Taken together, Nf1 might be directly involved in mediating molecular signal transduction from Inc to PKA signaling.

### Excessive memory functions in *inc* mutants are not restored via PKA manipulations

**Fig 7:**
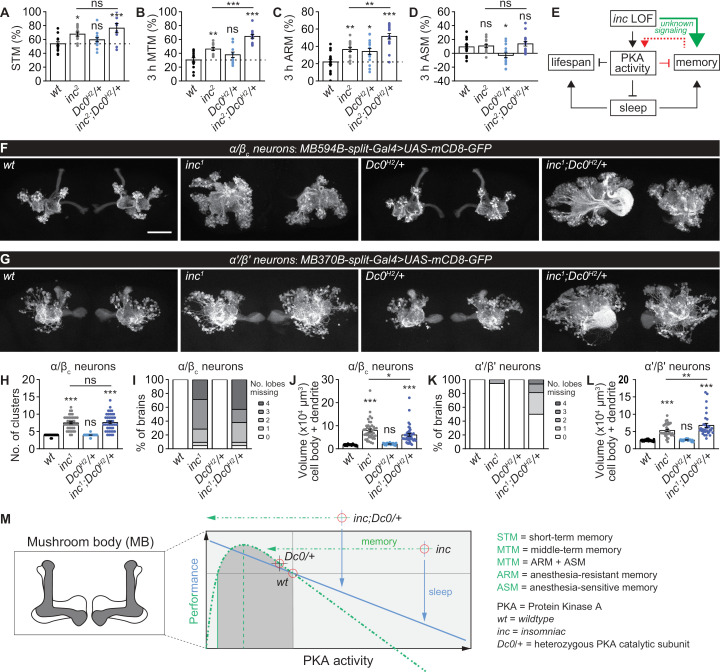
Excessive memory functions in *inc* mutants are not restored via PKA manipulations. (**A**–**D**) Olfactory memory tested immediately or 3 h after training for *wt*, *Dc0*^*H2*^/+ and *inc*^*2*^ with or without *Dc0*^*H2*^/+, including STM (**A**), MTM (**B**), ARM (**C**) and ASM (**D**). *n* = 9–15 for STM, 12–14 for MTM, and 16 for ARM and ASM. (**E**) A simplified model for roles of PKA signaling in coordinating memory, sleep and longevity. *inc* loss-of-function (LOF) is memory-promoting potentially through unknown signaling pathways, which is limited simultaneously by elevated PKA signaling. Thus, it is plausible that memory hyperfunction in *inc* mutants provokes adaptive higher PKA activity, which in turn suppresses memory functions. As a consequence, excessive PKA kinase activity provokes severe sleep deficits and compromised life span in *inc* mutants. The rescue effects of *Dc0*^*H2*^/+ on life span and its effects on further *inc*reasing the excessive memory of *inc* mutants might be either a direct role of PKA, or a consequence of the restored sleep. (**F** and **G**) Representative images of whole-mount brain expressing mCD8-GFP in subtypes of mushroom body α/β_c_ (**F**) or α′/β′ (**G**) Kenyon cells in *wt*/control, *Dc0*^*H2*^/+ and *inc*^*1*^ with or without *Dc0*^*H2*^/+ backgrounds. α/β_c_ neurons form discrete clusters in morphology, but not for α′/β′ neurons. Scale bar: 50 μm. (**H**–**L**) Statistics of the cluster number of α/β_c_ neurons (**H**), the number of lobes (**I** and **K**) and the volume of cell body and dendrites (**J** and **L**). *n* = 30–42 for **H** and **J**, 15–21 for **I**, and 32–36 for **L**. (**M**) Hypothesized model for the performance in sleep and memory controlled by PKA activity. Our data and previous studies suggest a bidirectional sleep regulation by PKA signaling. In contrast, memory is bidirectionally regulated likely only when PKA signaling is tuned within a *physiological* range. Severe and *pathological* PKA signaling reduction or increase is detrimental for memory performance. *inc* mutants seem to have higher setpoints for both memory and PKA activity. Restoring sleep to *inc* mutants by mildly titrating PKA signaling to half dose further promotes memory performance. *p < 0.05; **p < 0.01; ***p < 0.001; ns, not significant. Error bars: mean ±  SEM. Underlying data can be found in S7 Data.

Our findings that the intrinsic elevation of PKA signaling underlines the sleep and longevity phenotypes of *inc* mutants suggest that their excessive memory might also be rescued by *Dc0* heterozygosity. To directly test this hypothesis, we first confirmed the *Dc0*^*H2*^ heterozygosity rescue effects on sleep in the same fly cohort prepared for olfactory memory (S6A Fig). While *Dc0*^*H2*^ heterozygosity did not exhibit any significant effects in *wt*/control background, it further slightly increased STM (Fig 7A and S6B Fig) and significantly further promoted the higher 3 h MTM of *inc*^*2*^ mutants (Fig 7B and S6C Fig). The ARM component was also further increased by establishing *Dc0*^*H2*^ heterozygosity in *inc*^*2*^ mutants (Fig 7C). Notably, *Dc0*^*H2*^ heterozygous animals displayed elevated ARM and diminished ASM (Fig 7C and 7D), consistent with a previous study [75].

How might Inc interact with PKA signaling in the regulation of memory? Both Inc and PKA are widely expressed in the fly brain but enriched in the mushroom body [31,32,71]. As shown above, *inc* mutants exhibit gross mushroom body structural defects (Fig 5C, 5F, 5G and 5J). Furthermore, severe circuit defects in subtypes of mushroom body neurons in *inc* mutants, especially for the α/β core (α/β_c_) and α′/β′ Kenyon cells, have been previously reported [35]. In this regard, a simple hypothesis might be that the further increased memory performance of *inc* mutants by *Dc0* heterozygosity (Fig 7A–7E) could be due to a rescue of the mushroom body structural defects. To address this question, we expressed a GFP reporter in either α/β_c_ or α′/β′ Kenyon cells and evaluated the effects of Inc/PKA signaling in regulating the morphological properties of mushroom body subtype neurons. Consistent with previous findings [35], *inc* mutants showed an increased number of clusters of cell bodies, severely affected lobe structure, and an increased volume of cell bodies and dendrites of α/β_c_ neurons (Fig 7F and 7H–7J). While the cluster number phenotype remained unaffected (Fig 7H), the severe lobe structural phenotype was even more frequently triggered by *Dc0* heterozygosity (Fig 7I), while the cell body and dendritic volume phenotypes were slightly suppressed (Fig 7J). In addition to α/β_c_ neurons, *inc* mutants did almost never show any phenotype in the number of lobes of α′/β′ neurons, whose cell body and dendritic volume was also increased by *Dc0* heterozygosity (Fig 7G, 7K and 7L). Both lobe number and volume of cell bodies and dendrites of *inc* mutants were obviously further increased by *Dc0* heterozygosity (Fig 7G, 7K and 7L). These data suggest that the further increased memory performance of *inc* mutants with *Dc0* heterozygosity is unlikely to be achieved by restored mushroom body circuitry. Instead, the higher olfactory memory performance of *inc* mutants might be at least partially caused by overgrowth and abnormal arborization of these circuits. To a certain extent, the elevated PKA signaling might suppress mushroom body Kenyon cell overgrowth to constrain memory performance, and at the same time drive sleep loss in *inc* mutants. Regarding the functions of sleep, the substantially restored sleep in *inc* mutants by *Dc0* heterozygosity likely contributes to the memory benefits associated with the physiological functions of sleep.

Suppressing PKA signaling in the mushroom body by PKA.R1^BDK^ expression restored sleep, survival upon H_2_O_2_ treatment, and longevity in *inc* mutants (Figs 5L–5R, 6M and 6N). Comparing to *Dc0* heterozygosity (Fig 7A–7E), we wondered whether mushroom body-specific PKA.R1^BDK^ expression would regulate memory in a similar manner in *wt* and *inc* mutants. In addition, it is unclear how mushroom body-specific PKA signaling reduction affects the mushroom body structural phenotypes of *inc* mutants. To address these questions, we systematically examined memory (S6E–S6H Fig) and mushroom body structural phenotypes (S6I-S6K Fig) of animals expressing PKA.R1^GG^ (control) or PKA.R1^BDK^ driven by *VT30559-Gal4* in either *wt* or *inc* mutant background. We found that STM was not affected by mushroom body-specific PKA.R1^BDK^ expression (S6E Fig). However, while MTM was not affected in *wt* background, it was surprisingly decreased in *inc* mutant background with either PKA.R1^GG^ or PKA.R1^BDK^ expression (S6F Fig). This decrease in MTM is due to a clear reduction in ASM component (S6H Fig). Importantly, the ARM component was consistently increased in *inc* mutants and in PKA.R1^BDK^-expressing animals (S6G Fig). PKA.R1^BDK^ expression did not further increase the ARM of *inc* mutants (S6G Fig), however. Similar to the interaction between *inc* and *Dc0* heterozygosity, PKA.R1^BDK^ expression did not rescue the mushroom body structural phenotypes of *inc* mutants (S6I-S6K Fig).

These data suggest that mushroom body-specific PKA.R1^BDK^ expression partially mimicked *Dc0* heterozygosity in regulating mushroom body structure, sleep, life span and ARM component. The distinctions between these two genetic scenarios might derive from (1) additional circuits and tissues besides the mushroom body Kenyon cells being involved in dictating the effects of *Dc0* heterozygosity in memory and survival under H_2_O_2_ treatment; and (2) the different strength of PKA signaling reduction between these two different genetic PKA manipulations accounting for these behavioral differences. cAMP/PKA signaling has been shown to regulate sleep bidirectionally [28], while both severe increase and decrease of cAMP/PKA signaling are detrimental for memory (Fig 7M). Thus, it seems possible that *Dc0* heterozygosity triggers a level of PKA signaling optimal for restoring life expectancy but further promoting memory functions of *inc* mutants. In contrast, PKA.R1^BDK^ expression may result in much stronger PKA signaling reduction that is detrimental for memory of *inc* mutants (Fig 7M).

## Discussion

One of the major hypothesized functions of sleep is to restore brain plasticity from prior wakefulness, preparing the brain for subsequent episodes of learning and memory [6]. Consistently, learning and memory processes promote sleep for memory consolidation [10,37,76,77]. Furthermore, genetic and pharmacological sleep induction supports cognitive functions in health and disease [11,37,38,49,78]. Following this rationale, interfering with sleep, e.g., in genetic scenarios of reduced sleep, should attenuate the efficacy of memory functions. In this study, we started from our paradoxical observation that *inc* mutants, characterized by severely decreased sleep, display robustly elevated olfactory memory. We further found that *inc* LOF suppresses sleep through elevated PKA signaling in the mushroom body, which in turn constrains their excessive memory functions. These observations suggest a hyperfunction scenario on molecular, cellular, circuit and behavioral levels. This hyperfunction of *inc* mutants may stem from increased mushroom body neurogenesis and overproduction of Kenyon cells with arborization defects, as reported previously [35]. In direct consequence, the balance between the need of sleep and the strength of memory functions seems to be changed (Fig 7M). Brains lacking *inc* seemingly counteract this hyperfunction by elevating PKA signaling in the mushroom body. While this elevated PKA signaling restricts the memory hyperfunction, it comes at the cost of reduced sleep levels and shortened life span in *inc* mutants. Thus, our work might offer a mechanistic interpretation for the sleep phenotypes of *inc* mutants. These results may also hold broader relevance for understanding neurological disorders with developmental origin.

### *inc* LOF drives mushroom body overgrowth contributing to memory hyperfunction

It seems paradoxical that defective mushroom body morphology is coupled with excessive memory in *inc* mutants (Fig 7). Inc has been shown to be active during a specific time window of *Drosophila* brain development [35]. The overproduction of the mushroom body Kenyon cells with structural defects in *inc* mutants suggests that the mushroom body is the crucial neuron population from where Inc functions during mushroom body development to regulate both sleep and memory. Notably, we found that *Dc0*^*H2*^ heterozygosity further enhances the mushroom body structural phenotypes of *inc* mutants (Fig 7F–7L), but at the same time increases their excessive memory performance (Fig 7A–7E) and restores their sleep level (Fig 4B and S6A Fig). Thus, this overgrowth of mushroom body Kenyon cells might be causally related to the memory hyperfunction of *inc* mutants.

We currently lack a mechanistic insight into how this mushroom body overgrowth connects to memory hyperfunction and sleep deficits. Loss of *inc* generates more mushroom body Kenyon cells, which might enhance existing pathways or potentially establish novel circuits supporting excessive olfactory memory functions, and as a compensatory mechanism, elevated PKA signaling likely limits the mushroom body structural phenotypes and subsequently suppresses the memory hyperfunction. In addition, the over-production of Kenyon cells may also increase the number of odor-responsive Kenyon cells, leading to memory hyperfunction.

### How might *inc* LOF promote memory performance?

As discussed above, *inc* LOF exhibited enhanced memory, and downregulating PKA signaling in *inc* background further exacerbated this phenotype, suggesting that an elevated PKA signaling limits the memory hyperfunction of *inc* mutants. However, it is currently unknown how *inc* LOF triggers excessive olfactory memory in the first place. It appears likely that *inc* LOF promotes memory performance through unknown signaling pathways (Fig 7E).

While PKA signaling acting like a “brake” for higher memory performance in *inc* mutants (Fig 7A–7D, 7M and S6D Fig), what might be the unknown signaling pathways directly mediating the memory-promoting effects of *inc* mutants? As an adaptor protein for Cullin-3 ubiquitin E3 ligase, Inc might promote the degradation of a direct target of Cullin-3 ligase, whose accumulation due to impaired ubiquitination and degradation might be responsible for the memory-promoting effects of *inc* mutants. In this regard, a potential target of Inc/Cullin-3 complex might be the dopaminergic signaling involved in both sleep and memory regulation, as it was previously shown to be important in mediating the sleep phenotypes of *inc* mutants [32]. Furthermore, given that *inc* mutants have profound mushroom body circuit phenotypes, it will be important in the future to screen for the interactions between Inc and other factors involved in regulating mushroom body development. Indeed, mushroom body-specific expression of a gain-of-function Rho kinase (ROCK or Rok) promotes memory formation [79], and triggers mushroom body structural defects [80], similar to *inc* mutants. While potential effects of ROCK gain-of-function on sleep have not been addressed, *inc* LOF might promote excessive memory and mushroom body structural defects by modulating Ras/Raf/ROCK signaling.

### A potential hierarchical signaling cascade links Inc to PKA

Our data suggest that the elevated PKA signaling seems to constrain the excessive memory in *inc* mutants. What might be the exact action of Inc in regulating PKA signaling? The evolutionarily conserved nature of Inc as an adaptor of Cullin-3 E3 ligase for ubiquitination of target proteins suggests that PKA/Dc0 might be a direct target of Inc/Cullin-3. Indeed, *in vitro* studies suggest that PKA catalytic subunit is regulated through CHIP E3 ligase mediated ubiquitination and proteolysis [81]. However, there is no evidence for a role of Cullin-3 in PKA ubiquitination so far and Dc0 protein level does not seem to differ in *inc* mutants (Fig 5C and 5D). Given the elevated PKA kinase activity in *inc* mutants (Fig 5G–5I), and that their sleep phenotypes can be substantially rescued by suppressing PKA kinase activity with the expression of an active form of its regulatory subunit (Fig 5K–5S), it is more plausible that Inc indirectly regulates PKA activity through cAMP/PKA signaling regulators, for example Nf1 (Fig 3F), adenylate cyclase Rut and/or PKA regulatory subunits (Fig 3G and 3H). Thus, it will be interesting in the future to explore the direct Inc/Cullin-3 ubiquitination targets, which may contribute to the sleep deficits and memory hyperfunction of *inc* mutants.

### A Yin and Yang of cAMP/PKA signaling in integrating sleep with memory

cAMP/PKA signaling was among the first identified pathways controlling learning and memory [82]. Furthermore, cAMP/PKA signaling levels bidirectionally and negatively regulate sleep (Fig 7M) [28], consistent with our finding that the elevated PKA kinase activity of *inc* mutant directly drives their sleep deficits (Fig 5G–5S).

Notably, genetically and pharmacologically inducing sleep in the cAMP adenylate cyclase mutant *rutabaga* (which should suffer from reduced PKA activity) rescues their memory phenotypes [38]. Thus, lower cAMP/PKA signaling in *rutabaga* mutants might require excessive sleep for achieving a certain level of memory performance. In this regard, *Dc0* overexpression in the mushroom body suppresses memory at young age [29,83]. Conversely, a mild reduction in the protein level of Dc0 in *Dc0* heterozygotes prominently suppresses age-associated memory decline, while leaving memory unaffected at young age [29]. We here show that such genetic manipulation does not seem to provoke a major sleep phenotype in daily sleep amount in young *wt*/control animals (Figs 3A and 4A), while in *inc* background it specifically rescued the sleep defects (Figs 2A–2F and 3A–3E) and further increased memory performance (Fig 7A–7E). Different from *rutabaga* mutants whose changes of PKA activity might rather be outside the physiological range, *Dc0* heterozygosity might mimic physiologically relevant scenarios (Fig 7M), allowing for maintenance of longevity and memory (Fig 7E and S6D Fig).

Intriguingly, *Dc0* heterozygous animals are compromised in survival upon acute oxidative stress (Fig 6A), despite being ultimately longer-lived compared to *wt*/control animals upon normal aging (Fig 6B). Why would a reduction in PKA signaling promote longevity but fail to protect against oxidative stress? This may be explained by the differential regulation of downstream pathways by PKA in responding to acute stress versus chronic aging. Alternatively, PKA signaling might be differentially required for survival under acute oxidative stress and the chronic stress associated with aging. Indeed, acute H_2_O_2_ exposure directly activates PKA, followed by subsequent phosphorylation of substrate proteins *in vivo* [84], which might be critical for oxidative stress resistance. Interestingly, cAMP level, Dc0 protein level and PKA kinase activity in fly heads remain unchanged during early aging at age 20-day [29], whereas a significant reduction in Dc0 level is observed in 30-day-old fly wing neurons [85]. Thus, mushroom body cAMP/PKA signaling might become adverse for longevity and memory during aging.

The cAMP/PKA signaling pathway appears to regulate sleep, memory and survival in a spatiotemporal-specific manner. The sustained memory performance during aging and extended life span observed in *Dc0*^*H2*^ heterozygous animals seem to come at the cost of reduced resistance to acute oxidative stress. This delicate balance suggests a “Yin and Yang” perspective, reflecting the dual and context-dependent roles of cAMP/PKA signaling in mediating distinct cellular functions under varying circumstances.

PKA phosphorylates a wide range of targets, including critical synaptic proteins involved in neurotransmission and synaptic plasticity [86–88]. In mice, the AMP-activated protein kinase SIK3-mediated protein phosphorylation promotes sleep [89], while PKA suppresses sleep potentially by phosphorylating and inactivating SIK3 activity [90]. In SIK3 *Sleepy* mice, while their overall synaptic protein phosphorylation likely represents a signature of sleep need [89], its contribution to cognitive functions remains uncertain. In *inc* mutants, elevated PKA activity may induce a phosphorylation state driving wakefulness. Furthermore, *Dc0*^*H2*^ heterozygosity likely renormalizes the phosphorylation state and subsequently the level of sleep of *inc* mutants, which further supports even higher memory functions (Fig 7A–7E). Future studies will utilize quantitative proteomic and phosphoproteomic analyses in combination with sleep and memory screens to elucidate these phosphorylation states, providing insights into mechanisms underlying age-associated memory decline.

### Neurodevelopmental hyperfunction among neurological disorders

Collectively, by intensively studying the seemingly paradoxical *inc* short sleep mutants, we propose a cellular and behavioral hyperfunction scenario likely originating from the developmental overgrowth of mushroom body Kenyon cells, an essential brain center for sensory integration and sleep regulation in *Drosophila*. Our work identifies a molecular signaling cascade elicited by Inc in controlling PKA signaling, which tunes the balance between memory functions and the amount of sleep. While behavioral hyperfunction, coupled with sleep deficits and cognitive imbalances, mirrors hallmark traits of neurodevelopmental disorders such as autism [91, 92], the potential etiology to these disorders remains obscure. As Inc functions as an adaptor for Cullin-3 E3 ligase-mediated ubiquitination [31,36], and Cullin-3 mutations have been associated with autism spectrum disorder [93,94], our findings provide a potential mechanistic connection between neurodevelopmental hyperfunction and the etiology of autism.

## Materials and methods

### *Drosophila* stocks and maintenance

Flies were reared under standard laboratory conditions and raised on semi-defined medium (Bloomington recipe) under a 12/12 h light/dark cycle with 65% humidity at 25 °C. 5- to 8-day-old male flies were used for all experiments except aversive olfactory memory experiments in which mixed populations of both sexes were used. All fly strains were backcrossed to *w*^*1118*^ (*iso31*, BDSC#5905) background for at least six generations or retained in an already isogenized background. For the use of the two *inc* alleles in different experiments, due to the fact that *inc*^*2*^ mutants are in general healthier than *inc*^*1*^, *inc*^*2*^ flies were more frequently used than *inc*^*1*^, especially for Pavlovian aversive olfactory conditioning experiments, in which large number of flies are required. However, due to the UAS cassette in the P-element of *inc*^*2*^ mutants which drives *inc* expression in the presence of *Gal4* lines (Fig 1O) [31,35], Gal4/UAS binary expression system was only used in *inc*^*1*^ background. Most of the GABAergic signaling-related mutants used in sleep screening were from the Chemoconnectomics collection [95]. All original fly lines used in this study and their sources can be found within Table 1.

**Table 1:** Key resources used in this study.

REAGENT or RESOURCE	SOURCE	IDENTIFIER
Antibodies		
Mouse anti-BRP Nc82	Developmental Studies Hybridoma Bank	RRID: AB_2314866
Rabbit anti-GFP Alexa 488	Invitrogen	RRID: AB_221477
Mouse anti-FasII	Developmental Studies Hybridoma Bank	RRID: AB_528235
Mouse anti-FLAG	Sigma-Aldrich	RRID: AB_262044
Goat anti-mouse Alexa Fluor 488	Invitrogen	RRID: AB_2534069
Goat anti-mouse Alexa Fluor 647	Invitrogen	RRID: AB_2535804
Biological Samples		
*D. melanogaster: w* ^*1118*^	Bloomington *Drosophila* Stock Center	RRID: BDSC_5905
*D. melanogaster: Canton-S*	S. Sigrist lab	N/A
*D. melanogaster: mns*	Bloomington *Drosophila* Stock Center	RRID: BDSC_24149
*D. melanogaster: brp*^*c04298*^ (1xBRP)	Harvard Medical School	FlyBase: FBti0044871
*D. melanogaster: nf1* (*c00617*)	Bloomington *Drosophila* Stock Center	RRID: BDSC_10201
*D. melanogaster: Fbxl4* (*e02322*)	Bloomington *Drosophila* Stock Center	RRID: BDSC_18041
*D. melanogaster: homer* (*R102*)	Bloomington *Drosophila* Stock Center	RRID: BDSC_9564
*D. melanogaster: wake* (*EY02219*)	Bloomington *Drosophila* Stock Center	RRID: BDSC_15858
*D. melanogaster: fmn*	M. Freissmuth lab	FlyBase: FBal0197506
*D. melanogaster: inc*^*2*^ (*f00285*)	Bloomington *Drosophila* Stock Center	RRID: BDSC_18307
*D. melanogaster: inc* ^*1*^	N. Stavropoulos lab	FlyBase: FBal0266013
*D. melanogaster: aus* (*EY06350*)	Bloomington *Drosophila* Stock Center	RRID: BDSC_16396
*D. melanogaster: atg1* ^*EY07351*^	Bloomington *Drosophila* Stock Center	RRID: BDSC_16816
*D. melanogaster: Thor* ^*2*^	Bloomington *Drosophila* Stock Center	RRID: BDSC_9559
*D. melanogaster: S6K* ^*CC01583*^	Bloomington *Drosophila* Stock Center	RRID: BDSC_51563
*D. melanogaster: Drep-2* ^*ex13*^	S. Sigrist lab	FlyBase: FBal0300864
*D. melanogaster: Lrrk* ^*e03680*^	Bloomington *Drosophila* Stock Center	RRID: BDSC_85160
*D. melanogaster: Lk6* ^*2*^	Bloomington *Drosophila* Stock Center	RRID: BDSC_8707
*D. melanogaster: foxo* ^*Δ94*^	Bloomington *Drosophila* Stock Center	RRID: BDSC_42220
*D. melanogaster: Drep-1* ^*dICAD*^	S. Nagata lab	N/A
*D. melanogaster: Drep-4* ^*f02123*^	Harvard Medical School	FlyBase: FBal0181977
*D. melanogaster: Drep-4* ^*KG09839*^	Bloomington *Drosophila* Stock Center	RRID: BDSC_15217
*D. melanogaster: atg6* ^*1*^	R. Hiesinger lab	FlyBase: FBal0288277
*D. melanogaster: Rdl* ^*attP*^	Bloomington *Drosophila* Stock Center	RRID: BDSC_84569
*D. melanogaster: Gad1* ^*KG01955*^	Bloomington *Drosophila* Stock Center	RRID: BDSC_14174
*D. melanogaster: Dc0* ^*B3*^	M. Saitoe lab	FlyBase: FBal0033955
*D. melanogaster: Dc0* ^*H2*^	M. Saitoe lab	FlyBase: FBal0033960
*D. melanogaster: CalpA* ^*KG05080*^	Bloomington *Drosophila* Stock Center	RRID: BDSC_13868
*D. melanogaster: CalpB* ^*EY08042*^	Bloomington *Drosophila* Stock Center	RRID: BDSC_17422
*D. melanogaster: me31B* ^*k06607*^	Bloomington *Drosophila* Stock Center	RRID: BDSC_10635
*D. melanogaster: meng* ^*MB11376*^	Bloomington *Drosophila* Stock Center	RRID: BDSC_29914
*D. melanogaster: zfh1* ^*MB07519*^	Bloomington *Drosophila* Stock Center	RRID: BDSC_25351
*D. melanogaster: git* ^*ex10*^	S. Sigrist lab	FlyBase: FBal0320067
*D. melanogaster: syt1* ^*T77*^	Bloomington *Drosophila* Stock Center	RRID: BDSC_4377
*D. melanogaster: syt4* ^*EY12073*^	Bloomington *Drosophila* Stock Center	RRID: BDSC_20341
*D. melanogaster: endoA* ^*EP927*^	Bloomington *Drosophila* Stock Center	RRID: BDSC_24881
*D. melanogaster: Dap160* ^*EP2543*^	Bloomington *Drosophila* Stock Center	RRID: BDSC_19582
*D. melanogaster: rab3-GAP* ^*c04953*^	Bloomington *Drosophila* Stock Center	RRID: BDSC_85971
*D. melanogaster: GABA-B-R1* ^*attP*^	Bloomington *Drosophila* Stock Center	RRID: BDSC_84503
*D. melanogaster: GABA-B-R2* ^*attP*^	Bloomington *Drosophila* Stock Center	RRID: BDSC_84504
*D. melanogaster: GABA-B-R3* ^*attP*^	Bloomington *Drosophila* Stock Center	RRID: BDSC_84505
*D. melanogaster: vGat* ^*attP*^	Bloomington *Drosophila* Stock Center	RRID: BDSC_84586
*D. melanogaster: brp* [Pacman] (3xBRP)	S. Sigrist lab	FlyBase: FBal0296089
*D. melanogaster: arl8* ^*e00336*^	Bloomington *Drosophila* Stock Center	RRID: BDSC_17846
*D. melanogaster: αSnap* ^*f04776*^	Bloomington *Drosophila* Stock Center	RRID: BDSC_85684
*D. melanogaster: spn* ^*ex3.1*^	S. Sigrist lab	N/A
*D. melanogaster: unc13* ^*f07072*^	Bloomington *Drosophila* Stock Center	RRID: BDSC_19037
*D. melanogaster: cpx* ^*Δ2*^	Bloomington *Drosophila* Stock Center	RRID: BDSC_64252
*D. melanogaster: dbt* ^*EY02910*^	Bloomington *Drosophila* Stock Center	RRID: BDSC_19662
*D. melanogaster: Cul3* ^*EY11031*^	Bloomington *Drosophila* Stock Center	RRID: BDSC_20656
*D. melanogaster: bchs* ^*58*^	Bloomington *Drosophila* Stock Center	RRID: BDSC_9887
*D. melanogaster: Gat* ^*33-1*^	M. Freeman lab	FlyBase: FBal0375952
*D. melanogaster: relish* ^*EY08061*^	Bloomington *Drosophila* Stock Center	RRID: BDSC_20016
*D. melanogaster: dPC* ^*BG01252*^	Bloomington *Drosophila* Stock Center	RRID: BDSC_12461
*D. melanogaster: Dc0*^*BG*^ (*BG02142*)	Bloomington *Drosophila* Stock Center	RRID: BDSC_12752
*D. melanogaster: tau* ^*KO*^	Bloomington *Drosophila* Stock Center	RRID: BDSC_64782
*D. melanogaster: Gabat* ^*f01602*^	Bloomington *Drosophila* Stock Center	RRID: BDSC_85192
*D. melanogaster: rim* ^*EY12862*^	Bloomington *Drosophila* Stock Center	RRID: BDSC_21448
*D. melanogaster: aus* ^*MB07933*^	Bloomington *Drosophila* Stock Center	RRID: BDSC_25578
*D. melanogaster: acn* ^*tG4*^	Bloomington *Drosophila* Stock Center	RRID: BDSC_79328
*D. melanogaster: aplip1* ^*DG20707*^	S. Sigrist lab	FlyBase: FBal0218875
*D. melanogaster: mGluRA* (*112b*)	N. Naidoo lab	FlyBase: FBal0213218
*D. melanogaster: larp* ^*PL00175*^	Bloomington *Drosophila* Stock Center	RRID: BDSC_19407
*D. melanogaster: sra* ^*KG00335*^	Bloomington *Drosophila* Stock Center	RRID: BDSC_13093
*D. melanogaster: rolled* ^*sem*^	T. Raabe lab	FlyBase: FBal0014598
*D. melanogaster: liprin-α* ^*F3ex15*^	Bloomington *Drosophila* Stock Center	RRID: BDSC_8563
*D. melanogaster: liprin-α* ^*R60*^	Bloomington *Drosophila* Stock Center	RRID: BDSC_8561
*D. melanogaster: syd-1* ^*ex1.2*^	S. Sigrist lab	FlyBase: FBab0047210
*D. melanogaster: syd-1* ^*ex3.4*^	S. Sigrist lab	FlyBase: FBab0047211
*D. melanogaster: syd-1* ^*G9207*^	Bloomington *Drosophila* Stock Center	RRID: BDSC_32625
*D. melanogaster: nrx* ^*d08766*^	Bloomington *Drosophila* Stock Center	RRID: BDSC_19299
*D. melanogaster: dilp2* ^*1*^	Bloomington *Drosophila* Stock Center	RRID: BDSC_30881
*D. melanogaster: Dc0* ^*DN*^	Bloomington *Drosophila* Stock Center	RRID: BDSC_5282
*D. melanogaster: Dc0* ^*G4*^	Bloomington *Drosophila* Stock Center	RRID: BDSC_65501
*D. melanogaster: PKA.R1*^*c*^ (*c06969*)	Bloomington *Drosophila* Stock Center	RRID: BDSC_17792
*D. melanogaster: PKA.R1*^*MB*^ (*MB04145*)	Bloomington *Drosophila* Stock Center	RRID: BDSC_24569
*D. melanogaster: sss* (*EY04063*)	Bloomington *Drosophila* Stock Center	RRID: BDSC_16588
*D. melanogaster: cac* ^*H18*^	Bloomington *Drosophila* Stock Center	RRID: BDSC_42245
*D. melanogaster: mld* (*MB08505*)	Bloomington *Drosophila* Stock Center	RRID: BDSC_26381
*D. melanogaster: TH-Gal4*	Bloomington *Drosophila* Stock Center	RRID: BDSC_8848
*D. melanogaster: R24B11-Gal4*	Bloomington *Drosophila* Stock Center	RRID: BDSC_49070
*D. melanogaster: UAS-NaChBac*	T. Holmes lab	N/A
*D. melanogaster: UAS-Dc0.FLAG*	Bloomington *Drosophila* Stock Center	RRID: BDSC_35554
*D. melanogaster: UAS-PKA.SPARK*	F. Besse lab	FlyBase: FBti0235451
*D. melanogaster: Ca-α1T* (*del*)	Bloomington *Drosophila* Stock Center	RRID: BDSC_51994
*D. melanogaster: VT30559-Gal4*	Vienna *Drosophila* Resource Center	Cat# 206077
*D. melanogaster: UAS-mCD8-GFP*	Bloomington *Drosophila* Stock Center	RRID: BDSC_32184
*D. melanogaster: UAS-mCD8-GFP*	Bloomington *Drosophila* Stock Center	RRID: BDSC_32188
*D. melanogaster: UAS- PKA-R1* ^*GG*^	Bloomington *Drosophila* Stock Center	RRID: BDSC_35551
*D. melanogaster: UAS-PKA-R1* ^*BDK*^	Bloomington *Drosophila* Stock Center	RRID: BDSC_35550
*D. melanogaster: MB594B-split-Gal4*	Bloomington *Drosophila* Stock Center	RRID: BDSC_68255
*D. melanogaster: MB370B-split-Gal4*	Bloomington *Drosophila* Stock Center	RRID: BDSC_68319
Software and Algorithms		
Leica Application Suite X	Leica Microsystems	RRID: SCR_013673
ImageJ (Fiji)	http://fiji.sc	RRID: SCR_002285
GraphPad Prism	GraphPad Software	RRID: SCR_002798
MATLAB R2019a	MathWorks	RRID: SCR_001622

## Method details

### Aversive olfactory learning and memory

Pavlovian aversive olfactory conditioning was performed as previous reported [11,41]. Briefly, two aversive odors, 3-Octanol (OCT) and 4-methylcyclohexanol (MCH), served as behavioral cues (odors were diluted in mineral oil at a 1:100 ratio), and 120 V AC current electrical shocks were used as a behavioral reinforcer. Briefly, during one training session, around 100 naive flies were placed in a T-maze and exposed to the first odor (conditioned stimulus, CS^+^, MCH or OCT) paired with electric shocks (unconditioned stimulus, US) for 60 s. Afterwards, these flies were allowed to rest for 60 s followed by exposure to the second odor (CS^−^, OCT or MCH) without electric shocks for 60 s. During testing session, these flies were simultaneously exposed to CS ^+^ and CS^−^ at the two sides of the T-maze and they had 60 s to choose between the two odors. A reciprocal experiment, in which the second odor was paired with electric shocks as CS^+^, was performed. A performance index was calculated as the number of flies choosing CS^−^ minus the number of flies choosing CS^+^, divided by the total number of flies, and multiplied by 100 to get a percentage. The final performance index was the mean of the two performance indices from the two reciprocal experiments.


$$\text{Performance index }\left(\text{\%}\right)=\frac{{\text{CS}}^{-}-{\text{CS}}^{+}}{{\text{CS}}^{-}+{\text{CS}}^{+}}\times 100$$

STM was tested immediately after training, while 3 h MTM was tested 3 h after training. As a component of 3 h MTM, the 3 h ARM was separated from 3 h ASM by cold anesthesia. At 2 h 30 min after training, the trained flies received a cold anesthesia on ice for 90 s. 3h ARM was tested 30 min after the cold anesthesia, and 3 h ASM was calculated by subtracting 3 h ARM from 3 h MTM.


MTM=ARM +ASM

Odor avoidance was conducted similarly. Briefly, naive flies were placed in the choice position of the T-maze and allowed to choose between one of the two odors and air. The performance index was calculated as the number of flies choosing air minus the number of flies choosing the odor, divided by the total number of flies.

### Courtship conditioning

Courtship conditioning was performed similarly as previously reported [38]. Briefly, virgin *wt* and *inc*^*2*^ males were collected at eclosion (Day1) and individually housed until age 5-day before training (Day5). In parallel, virgin *wt* females and additional *wt* males were separated, collected and housed in groups until Day4. At Day4, in order to freshly pre-mate virgin *wt* females within 12 h prior training, 4-day-old *wt* virgin females were crossed to the additionally collected *wt* males. Pre-mated *wt* females were used at Day5 for training individually housed *wt* and *inc*^*2*^ males.

For training, individual *wt* or *inc*^*2*^ males were flipped into fresh food vials either with (trained) or without (naïve) a pre-mated *wt* female. The training process lasted for 30 min, and afterwards, trained and naïve males were transferred back to food vials and stayed individually before testing. Testing was performed within 30 min by using 3D-printed arenas (10 mm diameter and 4 mm height). The dimension of space provided for training was similar to the testing arenas. During testing, each trained or naïve male was placed with a pre-mated *wt* female in such an arena and their behaviors were recorded for more than 10 min.

Courtship behaviors were manually scored from the recorded 10-min videos and the total time a male spent on courting during testing was used to generate Courtship index (CI). Learning index (LI) was calculated as follows:


$$\text{Learning index }\left(\text{\%}\right)=\frac{{\text{CI}}_{\text{average naive}}-{\text{CI}}_{\text{trained}}}{{\text{CI}}_{\text{average naive}}}\times 100$$

In principle, freshly pre-mated *wt* females constantly reject courting attempts from males, and trained males will learn to reduce their courtship behaviors during the subsequently testing. Importantly in this regard, we did not observe any successful copulation of trained and naïve males for both *wt* and *inc*^*2*^ mutants. Thus, higher CI indicates longer courtship behavior and a reduction in CI of trained males compared to naïve males suggests successful courtship learning. Consistently, higher LI suggests stronger learning.

### Sleep measurements and genetic modifier sleep screen

Sleep experiments were performed exactly as previously reported [41]. Briefly, sleep of single male flies was measured by Trikinetics *Drosophila* Activity Monitors (DAM2) from Trikinetics (Waltham, MA) in 12/12 h light/dark cycle with 65% humidity at 25 °C. 3 ~ 4-day-old single male flies were loaded into Trikinetics glass tubes (5 mm inner diameter and 65 mm length) which have 5% sucrose and 2% agar in one side of the tube. Flies were entrained for at least 24 h and their locomotor activity was measured at 1 min interval. Due to entrainment to new environment, data from the first ~ 24 h were excluded. A period of immobility without locomotor activity counts lasting for at least 5 min was determined as sleep [22]. Sleep from multiple days of recordings was analyzed and averaged using the Sleep and Circadian Analysis MATLAB Program (SCAMP) [96].

To carry out genetic modifier sleep screen for the X-linked *inc* mutants, we crossed female virgin *inc* mutants to either *wt* as control or isogenized autosomal candidate mutants to introduce heterozygosity of candidate mutations to *inc* male hemizygous mutants. For each measurement or biological replicate, differences between *inc* mutant with or without heterozygous mutations were calculated and then different measurements or replicates were pooled to generate Fig 2. At least two biological replicates were performed for each candidate mutant. As *inc* mutants, especially *inc*^*2*^ mutants, show more profound sleep phenotypes during nighttime than during daytime, night P[wake] was specifically analyzed for the genetic modifier screen in Fig 2 so as to show major effects of different candidate heterozygous mutants on night sleep quality. For other P[wake] analysis, especially that *Dc0* heterozygosity has similar effects between day and night for *inc* mutants, daytime and nighttime were averaged for simplicity in main figures. Detailed daytime and nighttime sleep parameters were separately shown in supplementary figures (S1–S6 Figs).

### Climbing assay

Climbing assay was carried out as previous described [41]. Briefly, flies were sorted into groups of 10 flies after eclosion and aged to 5-days old. During testing, flies were gently transferred to the testing tubes (2.1 cm diameter) and allowed to rest for at least 5 min. Afterward, both *wt* and *inc* mutant flies were simultaneously and quickly tapped down to the bottom. Flies reacted to the tapping and started to climb, and this process was visualized and videotaped.

### Whole-mount brain immunostaining

Whole-mount brain immunostaining was performed similar to previous report [11,41]. Brains of 5-day old male flies were used for all staining experiments. Flies were kept on ice for 1–2 min and then dissected in ice-cold Ringer’s solution (130 mM NaCl, 5 mM KCl, 2 mM MgCl_2_, 2 mM CaCl_2_, 5 mM HEPES, 36 mM Sucrose, pH =  7.3). Dissected brains were fixed immediately in 4% Paraformaldehyde (w/v) in PBS for 30 min on an orbital shaker at room temperature. Samples were washed in 0.7% Triton-X (v/v) in PBS (0.7% PBST) for 20 min ×  3 times, followed by blocking with 10% normal goat serum (v/v) in 0.7% PBST for at least 2 h at room temperature on shaker. After overnight antibody (rabbit anti-GFP conjugated with Alexa 488, 1:500; Nc82, 1:50; anti-FLAG, 1:200; anti-FasII, 1:200) incubation at 4 °C in darkness, brains were washed in 0.7% PBST for 30 min ×  6 times at room temperature. Afterwards, for GFP Alexa 488 staining, brains were directly mounted on microscope slides in Vectashield and kept in a dark place at 4 °C before being scanned. For Nc82, FLAG and FasII staining, overnight secondary antibody (Alexa 488 or Cy5, 1:300) incubation at 4 °C in darkness followed by 6 times washing in 0.7% PBST was performed before mounting. Additional antibody information can be found within Table 1.

### Confocal microscopy and image analysis

Whole-mount adult brain samples were imaged on a Leica TCS SP8 confocal microscope from Leica Microsystems, and images were obtained using the Leica Application Suite X. For analyzing PKA SPARK intensity and puncta, the mushroom body lobes were imaged using a 63× 1.40 NA oil objective with a voxel size of 0.2405 µm ×  0.2405 µm ×  1 µm at a speed of 400 Hz. For morphology analysis, mushroom body lobes and calyx were imaged using a 40 × 1.30 NA oil objective with a voxel size of 0.3788 µm ×  0.3788 µm ×  1 µm. All the parameters were kept constant for the same set of experiments. Image stacks were processed and analyzed with Fiji (https://fiji.sc/).

### Mushroom body calyx and cell body volume analysis

The quantification of calyx volume was performed semi-automatically via a custom-written Fiji Macro, with steps briefly described in the following. Selected stacks containing regions of cell bodies and dendritic processes of the calyx were converted into binary images after background subtraction, image smoothing, and thresholding processes, and then the volumes were measured using the Plugin Voxel counter.

### Dc0-FLAG and PKA SPARK analysis

For Dc0-FLAG and PKA SPARK experiments, imaging stacks of mushroom body lobes were converted to two-dimensional images using max Z-projection. The average gray pixel value of the Dc0-FLAG and PKA SPARK was measured within a ROI of mushroom body lobes segmented automatically after background subtraction, image filtering, and thresholding processes in ImageJ (Fiji). The Dc0-FLAG and SPARK puncta were detected using “Find Maxima”.

### Oxidative stress survival assay

Male flies were sorted into a population of 45–50 flies per vial within 2 days post-eclosion. One day after sorting, the flies were transferred into a vial (25 mm diameter) containing 4 layers of filter paper soaked with 1.4 ml 5% sucrose solution with 2% H_2_O_2_. Flies were transferred into new vials with freshly prepared filter papers and solution every other day. The numbers of deaths were counted and recorded daily at the same time of the day. Comparison of survival curves was performed with GraphPad Prism using Log-rank analysis.

### Longevity

Longevity experiments were carried out exactly as previously reported [11]. Briefly, male flies were sorted into a population of approximately 20–25 flies per vial/replicate at the age of 2 days. To reduce the variability in life span, a few different cohorts of flies were used for each experiment. The longevity flies were regularly transferred onto fresh food every second day or over the weekend and the number of dead flies in each vial was recorded at each time of transfer until the death of the last fly of a replicate. Comparison of longevity curves was performed with GraphPad Prism using Log-rank analysis.

### Statistics

Statistics was performed as previously described [41]. GraphPad Prism 6 was used to perform most statistics and figures were generated using both Adobe Illustrator and Prism. Student *t* test was used for comparison between two groups and one-way ANOVA with Tukey’s post hoc tests were used for multiple comparisons between multiple groups (≥3). For mushroom body lobe analysis (Fig 7I, 7K and S6J, S6K Fig), Kruskal-Wallis test with Dunn’s multiple comparisons was used. Additional software information can be found within Table 1.

## Supporting information

S1 Fig Courtship conditioning and locomotor activity of inc mutants. (**A**) Courtship index for naïve and trained *wt* and *inc^2^* males. *n* = 28–31. (**B**) Learning index for *wt* and *inc*^*2*^ males. *n* = 28–30. (**C**–**F**) Daytime and nighttime sleep structure of *inc*^*1*^ and *inc*^*2*^ flies averaged from measurements over 2–4 days, including daily sleep amount (**C**), sleep latency at ZT0 (**D**), P[doze] (**E**) and P[wake] (**F**). *n* = 69–96. (**G**–**J**) Total daily locomotor count (**G**) and wake time (**H**) for calculating “*Activity per wake minute*” (**I**). “*Activity per active minute*” (**J**) was calculated as the average activity for each minute with at least one locomotor activity count. The locomotor index “*Activity per active minute*” was further used in this study. *n* = 69–96. *p < 0.05; **p < 0.01; ***p < 0.001; ns, not significant. Error bars: mean ±  SEM. Underlying data can be found in S1 Data Sheet.
(EPS)

S2 Fig Modulating the sleep phenotypes of inc mutants by Cullin-3, 1xBRP, and GABAergic signaling. (**A**) Daytime and nighttime sleep structure of *wt*, *Cul-3*/+ and 1xBRP flies averaged from measurements over 2–4 days, including daily sleep amount, sleep latency at ZT0 and ZT12, P[doze], P[wake] and locomotor index. *n* = 40–111. (**B**) Daytime and nighttime sleep structure of *inc*^*2*^ with either *Cul-3*/+ or 1xBRP averaged from measurements over 2–4 days, including daily sleep amount, sleep latency at ZT0 and ZT12, P[doze], P[wake] and locomotor index. *n* = 30–51. (**C**) Daytime and nighttime sleep structure of *wt*, *GABA-B-R1*^*attP*^/+ (*B-R1*/+) and *vGat*^*attP*^/+ flies averaged from measurements over 2–4 days, including daily sleep amount, sleep latency at ZT0 and ZT12, P[doze], P[wake] and locomotor index. *n* = 40–47. (**D**) Daytime and nighttime sleep structure of *inc*^*2*^ with either *GABA-B-R1*^*attP*^/+ (*B-R1*/+) or *vGat*^*attP*^/+ averaged from measurements over 2–4 days, including daily sleep amount, sleep latency at ZT0 and ZT12, P[doze], P[wake] and locomotor index. *n* = 30–51. *p < 0.05; **p < 0.01; ***p < 0.001; ns, not significant. Error bars: mean ±  SEM. Underlying data can be found in S1 Data Sheet.
(EPS)

S3 Fig cAMP/PKA signaling mediates the sleep phenotypes of inc mutants. (**A**) Daytime and nighttime sleep structure of *wt*, *Dc0*^*B3*^/+ and *Dc0*^*H2*^/+ flies averaged from measurements over 2–4 days, including daily sleep amount, sleep latency at ZT0, P[doze], P[wake] and locomotor index. *n* = 70–76. (**B**) Daytime and nighttime sleep structure of *inc*^*1*^ with either *Dc0*^*B3*^/+ or *Dc0*^*H2*^/+ averaged from measurements over 2–4 days, including daily sleep amount, sleep latency at ZT0, P[doze], P[wake] and locomotor index. *n* = 57–66. (**C**) Daytime and nighttime sleep structure of *inc*^*2*^ with either *Dc0*^*B3*^/+ or *Dc0*^*H2*^/+ averaged from measurements over 2–4 days, including daily sleep amount, sleep latency at ZT0, P[doze], P[wake] and locomotor index. *n* = 73–76. (**D**) Daytime and nighttime sleep structure of *wt*, *Dc0*^*BG*^/+ and *inc*^*1*^ with or without *Dc0*^*BG*^/+ averaged from measurements over 2–4 days, including daily sleep amount, sleep latency at ZT0, P[doze], P[wake] and locomotor index. *n* = 40–71. (**E**) Daytime and nighttime sleep structure of *wt* and *inc*^*1*^ with either *Dc0*^*DN*^/+ or *Dc0*^*G4*^/+ averaged from measurements over 2–4 days, including daily sleep amount, sleep latency at ZT0, P[doze], P[wake] and locomotor index. *n* = 32–48. (**F**) Daytime and nighttime sleep structure of *wt*, *nf1*/+ and *inc*^*2*^ with or without *nf1*/+ averaged from measurements over 2–4 days, including daily sleep amount, sleep latency at ZT0, P[doze], P[wake] and locomotor index. *n* = 62–80. (**G**) Daytime and nighttime sleep structure of *wt*, *PKA.R1*^*c*^/+ and *PKA.R1*^*MB*^/+ flies averaged from measurements over 4 days, including daily sleep amount, sleep latency at ZT0, P[doze], P[wake] and locomotor index. *n* = 58–63. (**H**) Daytime and nighttime sleep structure of *inc*^*1*^ with either *PKA.R1*^*c*^/+ or *PKA.R1*^*MB*^/+ averaged from measurements over 4 days, including daily sleep amount, sleep latency at ZT0, P[doze], P[wake] and locomotor index. *n* = 61–67. (**I**–**O**) Absolute difference in sleep of *inc* mutants provoked by heterozygosity of *Dc0* (**I**–**L**), *nf1* (**M**) and *PKA.R1* (**N** and **O**) alleles. *n* =  40–80. *p < 0.05; **p < 0.01; ***p < 0.001; ns, not significant. Error bars: mean ±  SEM. Underlying data can be found in S1 Data Sheet.
(EPS)

S4 Fig The effects of Dc0 heterozygosity on promoting sleep are specific to inc mutants. (A–L) Daytime and nighttime sleep structure of *Dc0^H2^*/+ in control (**A**), *inc^2^* (**B**), *mns* (**C**), *sss* (**D**), 1xBRP (**E**), *cac*^*H18*^ (**F**), *wake* (**G**), *mld* (**H**), *TH>NaChBac* (**I**), *R24B11 > NaChBac* (**J**), *mGluRA* (**K**) and *Ca-*α*1T* (**L**) backgrounds, averaged from measurements over 2–4 days, including daily sleep amount, sleep latency at ZT0 and ZT12, P[doze], P[wake] and locomotor index. *n* = 48–52 for **A**, 32–38 for **B**, 48–56 for **C**, 47–72 for **D**, 48–51 for **E**, 48 for **F**, 54–56 for **G**, 29–48 for **H**, 32–48 for **I** and **J**, 39–47 for **K**, and 48 for **L**. *p < 0.05; **p < 0.01; ***p < 0.001; ns, not significant. Error bars: mean ± SEM. Underlying data can be found in S1 Data Sheet.
(EPS)

S5 Fig Mushroom body-specific PKA signaling suppression rescues the sleep deficits of inc mutants. (**A**) Sleep structure of *VT30559-Gal4*/*+*, *UAS-PKA. R1*^*GG*^/+ and *VT30559 > PKA.R1*^*GG*^ flies averaged from measurements over 3–4 days, including sleep profile plotted in 30-min bins, daily sleep amount, P[doze], P[wake] and locomotor index. *n* = 39–48. (**B**) Sleep structure of *VT30559-Gal4*/+, *UAS-PKA.R1*^*GG*^/+ and *VT30559 > PKA.R1*^*GG*^ flies in *inc*^*1*^ mutant background averaged from measurements over 3 days, including sleep profile plotted in 30-min bins, daily sleep amount, P[doze], P[wake] and locomotor index. *n* = 30–32. (**C**) Daytime and nighttime sleep structure from measurements over 4 days of flies expressing non-functional (GG) or active (BDK) PKA regulatory subunit in the mushroom body driven by *VT30559-Gal4* in *wt*/control background, including daily sleep amount, sleep latency at ZT0, P[doze], P[wake] and locomotor index. *n* = 76. (**D**) Daytime and nighttime sleep structure from measurements over 4 days of flies expressing non-functional (GG) or active (BDK) PKA regulatory subunit PKA.R1 in the mushroom body driven by *VT30559-Gal4* in *inc*^*1*^ mutant background, including daily sleep amount, sleep latency at ZT0, P[doze], P[wake] and locomotor index. *n* = 83–85. (**E**) Daytime and nighttime sleep structure from measurements over 2–4 days of flies expressing non-functional (GG) or active (BDK) PKA regulatory subunit PKA.R1 in the mushroom body driven by *VT30559-Gal4* in 1xBRP background, including daily sleep amount, sleep latency at ZT0, P[doze], P[wake] and locomotor index. *n* = 27–48. *p < 0.05; **p < 0.01; ***p < 0.001; ns, not significant. Error bars: mean ±  SEM. Underlying data can be found in S1 Data Sheet.
(EPS)

S6 Fig Mushroom body-specific PKA suppression partially mimics Dc0 heterozygosity. (**A**) Daily sleep amount of *inc^2^* with or without *Dc0^H2^*/+. The *inc*^*2*^;*Dc0*^*H2*^/+ animals here were created by genetic combination for olfactory memory, while other identical results were achieved by a single round of crosses (also see Materials and methods). *n* = 12. (**B** and **C**) Absolute difference in STM (**B**) and MTM (**C**) of *inc* mutants provoked by *Dc0* heterozygosity. *n* =  9–12. (**D**) Detailed model derived from the mechanistic link between Inc and PKA signaling in regulating sleep, memory and survival, taking mushroom body overgrowth phenomenon into consideration. (**E**–**H**) Olfactory memory tested immediately or 3 h after training for *Ctrl* (*VT30559-Gal4*/+), *GG.*, *inc*^*1*^*;GG.*, *BDK* and *inc*^*1*^;*BDK.*, including STM (**E**), MTM (**F**), ARM (**G**) and ASM (**H**). *n* = 8–10 for STM, 12–16 for MTM, and 11 for ARM and ASM. (**I**) Representative images of whole-mount brain staining against FasII in *GG.*, *inc*^*1*^*;GG.*, *BDK* and *inc*^*1*^;*BDK* animals. Scale bar: 20 μm. (**J** and **K**) Statistics of mushroom body lobe structural (**J**) and midline extension phenotypes (**K**). *n* = 15. *p <  0.05; **p < 0.01; ***p < 0.001; ns, not significant. Error bars: mean ± SEM. Underlying data can be found in S1 Data Sheet.
(EPS)

S1 Video Normal climbing ability in *inc* mutants. (MOV)

S1 Data Data underlying Fig 1. (XLSX)

S2 Data Data underlying Fig 2. (XLSX)

S3 Data Data underlying Fig 3. (XLSX)

S4 Data Data underlying Fig 4. (XLSX)

S5 Data Data underlying Fig 5. (XLSX)

S6 Data Data underlying Fig 6. (XLSX)

S7 Data Data underlying Fig 7. (XLSX)

S1 Data Sheet Data underlying S1–S6 Figs. (XLSX)

## Abbreviations:

ARM: Anesthesia-resistant memory
ASM: Anesthesia-sensitive memory
cAMP: cyclic adenosine 3′, 5′-monophosphate
MCH: 4-methylcyclohexanol
MTM: Middle-term memory
Nf1: Neurofibromatosis-1
OCT: 3-Octanol
PKA: Protein Kinase A
Rut: Rutabaga
STM: Short-term memory
*wt*: wild-type
Dc0: PKA, catalytic subunit;
*inc*: , insomniac;
MB,: Mushroom body;
LOF,: Loss-of-function;
*mns*: , minisleep;
*fmn*: , fumin;
*sss*: , sleepless;
*wake*: , wide awake;
*aus*: , argus.
