# Supplementary material for: Enhanced memory despite severe sleep loss in Drosophila insomniac mutants
Source: PLoS Biol. 2025 Mar 20;23(3):e3003076. doi: 10.1371/journal.pbio.3003076 (PMC12135902; doi:10.1371/journal.pbio.3003076)
Supplement: Peer review history

# Author comment: Author response to Decision Letter 0

**DOI:** 10.1371/journal.pbio.3003076.r001

3 Feb 2025

# Editor report: Decision Letter 0

**DOI:** 10.1371/journal.pbio.3003076.r002

**Authors:** Lucas Smith

11 Feb 2025

Dear Dr Sigrist, 

Thank you for submitting your manuscript entitled "Enhanced memory despite severe sleep loss in Drosophila insomniac mutants" for consideration as a Research Article by PLOS Biology. We understand that your study has gone through several rounds of peer review at Current Biology, where you have addressed the technical issues raised by the reviewers - but where you are reaching an impasse with one of the reviewers regarding the presentation and semantic descriptions of the data. 

Your manuscript, the previous rounds of review, and your response(s) to reviewers have now been evaluated by the PLOS Biology editorial staff as well as by an academic editor with relevant expertise, under our portable peer review policy. After this discussion, and based on our academic editor's assessment, we agree that the technical issues have been addressed and we are satisfied by your responses to the most recent points raised by the critical reviewer from Current Biology. We therefore think that your manuscript would be suitable for publication at PLOS Biology without further revisions. However, before we can editorially accept your paper, we need you to complete the metadata and provide any underlying data and code. 

To complete your metadata please login to Editorial Manager where you will find the paper in the 'Submissions Needing Revisions' folder on your homepage. Please click 'Revise Submission' from the Action Links and complete all additional questions in the submission questionnaire. 

**IMPORTANT: 

--Please be sure to fill out all relevant sections (such as financial disclosures, competing interests, data availability, etc) and to follow the instructions carefully - as we will need these details before we can accept your study. 

--Also, below my signature I have included the details of our data and code policies - and I ask that you carefully look through these and make sure to supply all relevant underlying data, as that will also be needed prior to acceptance. 

--Additionally, I have reached out to the editors from Current Biology, to confirm the review history with their journal but have yet to hear back from them. However, in parallel, could you forward us the most recent decision letter from Current Biology? That will help us confirm the validity of the provided reports. You can forward the previous decision letter to my inbox at Lsmith@plos.org. 

Once your full submission is complete, your paper will undergo a series of checks by our operations team who may flag any issues - and then I will take a look to make sure that the study complies with our data availability policy, etc - before hopefully accepting the paper for publication. 

To provide the metadata for your submission and upload the underlying data, please Login to Editorial Manager (https://www.editorialmanager.com/pbiology) by Feb 17 2025 11:59PM. 

Feel free to email us at plosbiology@plos.org if you have any queries relating to your submission or if you need a bit of extra time. 

Kind regards,

Luke

Lucas Smith, Ph.D.

Senior Editor

PLOS Biology

lsmith@plos.org

DATA AND CODE POLICIES: 

DATA POLICY: You may be aware of the PLOS Data Policy, which requires that all data be made available without restriction: http://journals.plos.org/plosbiology/s/data-availability. For more information, please also see this editorial: http://dx.doi.org/10.1371/journal.pbio.1001797

Note that we do not require all raw data. Rather, we ask that all individual quantitative observations that underlie the data summarized in the figures and results of your paper be made available in one of the following forms:

1) Supplementary files (e.g., excel). Please ensure that all data files are uploaded as 'Supporting Information' and are invariably referred to (in the manuscript, figure legends, and the Description field when uploading your files) using the following format verbatim: S1 Data, S2 Data, etc. Multiple panels of a single or even several figures can be included as multiple sheets in one excel file that is saved using exactly the following convention: S1_Data.xlsx (using an underscore).

2) Deposition in a publicly available repository. Please also provide the accession code or a reviewer link so that we may view your data before publication. 

>>>Regardless of the method selected, please ensure that you provide the individual numerical values that underlie the summary data displayed your figures, as they are essential for readers to assess your analysis and to reproduce it. 

NOTE: the numerical data provided should include all replicates AND the way in which the plotted mean and errors were derived (it should not present only the mean/average values).

>>>Please also ensure that figure legends in your manuscript include information on where the underlying data can be found, and ensure your supplemental data file/s has a legend.

>>>Please ensure that your Data Statement in the submission system accurately describes where your data can be found.

CODE POLICY Per journal policy, if you have generated any custom code during the course of this investigation, please make it available without restrictions. Please ensure that the code is sufficiently well documented and reusable, and that your Data Statement in the Editorial Manager submission system accurately describes where your code can be found. 

Please note that we cannot accept sole deposition of code in GitHub, as this could be changed after publication. However, you can archive this version of your publicly available GitHub code to Zenodo. Once you do this, it will generate a DOI number, which you will need to provide in the Data Accessibility Statement (you are welcome to also provide the GitHub access information). See the process for doing this here: https://docs.github.com/en/repositories/archiving-a-github-repository/referencing-and-citing-content

BLOT AND GEL REPORTING REQUIREMENTS: We require the original, uncropped and minimally adjusted images supporting all blot and gel results reported in an article's figures or Supporting Information files. We will require these files before a manuscript can be accepted so please prepare and upload them now. Please carefully read our guidelines for how to prepare and upload this data: https://journals.plos.org/plosbiology/s/figures#loc-blot-and-gel-reporting-requirements

# Author comment: Author response to Decision Letter 1

**DOI:** 10.1371/journal.pbio.3003076.r003

14 Feb 2025

# Editor report: Decision Letter 1

**DOI:** 10.1371/journal.pbio.3003076.r004

**Authors:** Lucas Smith

19 Feb 2025

Dear Dr Sigrist,

Thank you again for the submission of your Research Article "Enhanced memory despite severe sleep loss in Drosophila insomniac mutants" which has been revised in response to reviews from Current Biology, for publication in PLOS Biology - and thank you also for now providing the relevant metadata and underlying data for your study. On behalf of my colleagues and the Academic Editor, Paul Shaw, I am pleased to say that we think the paper has adequately addressed the points raised in the previous rounds of review and that it meets our standards for publication. Therefore, we can in principle accept your manuscript for publication, provided you address any remaining formatting and reporting issues. These will be detailed in an email you should receive within 2-3 business days from our colleagues in the journal operations team; no action is required from you until then. Please note that we will not be able to formally accept your manuscript and schedule it for publication until you have completed any requested changes.

Please take a minute to log into Editorial Manager at http://www.editorialmanager.com/pbiology/, click the "Update My Information" link at the top of the page, and update your user information to ensure an efficient production process.

PRESS

We frequently collaborate with press offices. If your institution or institutions have a press office, please notify them about your upcoming paper at this point, to enable them to help maximise its impact. If the press office is planning to promote your findings, we would be grateful if they could coordinate with biologypress@plos.org. If you have previously opted in to the early version process, we ask that you notify us immediately of any press plans so that we may opt out on your behalf.

We also ask that you take this opportunity to read our Embargo Policy regarding the discussion, promotion and media coverage of work that is yet to be published by PLOS. As your manuscript is not yet published, it is bound by the conditions of our Embargo Policy. Please be aware that this policy is in place both to ensure that any press coverage of your article is fully substantiated and to provide a direct link between such coverage and the published work. For full details of our Embargo Policy, please visit http://www.plos.org/about/media-inquiries/embargo-policy/.

Thank you again for choosing PLOS Biology for publication and supporting Open Access publishing. We look forward to publishing your study. 

Sincerely, 

Luke

Lucas Smith, Ph.D.

Senior Editor

PLOS Biology

lsmith@plos.org
